# Involvement of two uptake mechanisms of gold and iron oxide nanoparticles in a co-exposure scenario using mouse macrophages

**DOI:** 10.3762/bjnano.8.239

**Published:** 2017-11-14

**Authors:** Dimitri Vanhecke, Dagmar A Kuhn, Dorleta Jimenez de Aberasturi, Sandor Balog, Ana Milosevic, Dominic Urban, Diana Peckys, Niels de Jonge, Wolfgang J Parak, Alke Petri-Fink, Barbara Rothen-Rutishauser

**Affiliations:** 1 Adolphe Merkle Institute, Université de Fribourg, Chemin des Verdiers 4, CH 1700, Fribourg, Switzerland; 2 CIC Biomagune, Miramon Ibilbidea 182, 20014 Donostia, Gipuzkoa, San Sebastian, Spain; 3 Department of Biophysics, CIPMM Geb. 48, Saarland University, 66421 Homburg/Saar, Germany; 4 INM - Leibniz Institute for New Materials, Campus D2 2, 66123 Saarbrücken, Germany; 5 Fachbereich Physik, Philipps Universität Marburg, Renthof 7, 35037 Marburg, Germany

**Keywords:** co-exposure, endocytosis, live cell imaging, nanoparticles, quantitative microscopy

## Abstract

Little is known about the simultaneous uptake of different engineered nanoparticle types, as it can be expected in our daily life. In order to test such co-exposure effects, murine macrophages (J774A.1 cell line) were incubated with gold (AuNPs) and iron oxide nanoparticles (FeO*_x_*NPs) either alone or combined. Environmental scanning electron microscopy revealed that single NPs of both types bound within minutes on the cell surface but with a distinctive difference between FeO*_x_*NPs and AuNPs. Uptake analysis studies based on laser scanning microscopy, transmission electron microscopy, and inductively coupled plasma optical emission spectrometry revealed intracellular appearance of both NP types in all exposure scenarios and a time-dependent increase. This increase was higher for both AuNPs and FeO*_x_*NPs during co-exposure. Cells treated with endocytotic inhibitors recovered after co-exposure, which additionally hinted that two uptake mechanisms are involved. Cross-talk between uptake pathways is relevant for toxicological studies: Co-exposure acts as an uptake accelerant. If the goal is to maximize the cellular uptake, e.g., for the delivery of pharmaceutical agents, this can be beneficial. However, co-exposure should also be taken into account in the case of risk assessment of occupational settings. The demonstration of co-exposure-invoked pathway interactions reveals that synergetic nanoparticle effects, either positive or negative, must be considered for nanotechnology and nanomedicine in particular to develop to its full potential.

## Introduction

Over the past two decades, improvements in nanomaterial research were followed by a rise of nanotechnology-containing products [1]. Numerous contemporary developments in consumer products (e.g., food additives, cosmetics and sporting equipment), environmental remediation, medicine and information technology heavily rely on innovative nanomaterial-driven industrial technology [2]. A comprehensive groundwork has been laid to assess the possible adverse effects of nanotechnology on human health. In particular, a full understanding of how such engineered nanoparticles (NPs), i.e., objects with all three spatial dimensions below 100 nm [3], interact with mammalian cells is paramount to develop diagnostic and therapeutic applications [4]. There is convincing evidence that the physico-chemical properties of NPs, such as size, shape, material, and surface functionalization, strongly influence uptake and intracellular trafficking of NPs [5–7]. The uptake of NPs occurs via endocytotic pathways, which can be subdivided into macro- and microscale processes [8]. The first, phagocytosis, involves the ingestion of large particles: NPs or agglomerates typically larger than 0.25 μm in diameter. The second, pinocytosis, includes micro- and macropinocytosis, clathrin-, and caveolin-mediated endocytosis, and clathrin- and caveolin-independent endocytosis, involving the ingestion of fluid, molecules, and NPs via small vesicles (<0.15 µm in diameter) [9]. Although NPs have been shown to be taken up by the cells mainly by pinocytotis [9], many factors have been shown to influence the interaction with cells such as cell type [10–11], surface charge [12–13] and NP size [14–15]. Clathrin- and caveolin-mediated endocytosis are the main uptake mechanisms for PEGylated AuNPs (15 nm) into A549 cells, whereas non PEGylated, citrate-stabilized AuNPs were mainly taken up by micropinocytosis [16], potentially reflecting different agglomeration states. The uptake mechanism for one and the same NP into different cell types may even vary [10]. For instance, the uptake of fetal bovine serum-treated titanium dioxide NPs (TiO_2_NP) into A549 and H1299 cells, two human lung cell lines, is different, and it was shown that the uptake of TiO_2_NP in A549 cells involves a clathrin-dependent pathway, whereas in H1299 a caveolae- and clathrin-independent pathway was observed [17]. Another uptake pathway for NP has been described recently by Freese et al., who observed AuNPs in the size range of 23–73 nm to enter microvascular endothelial cells via flotillin-mediated uptake [18]. Flotillin-mediated uptake was also observed for silica NPs in lung epithelial and endothelial cells [19].

The various uptake pathways have one aspect in common: The internalized NP is ultimately located in an intracellular vesicle. Endocytosis of NPs by cells results in the localisation of the particles first in early endosomes, then late endosomes, which finally fuse with lysosomes [20]. The particular uptake pathway determines the time it takes until the NPs are present in lysosomal compartments [15]. Whole-cell electron microscopy experiments revealed that the AuNPs were localised in their membranes rather than in the cores of the vesicles [21–22]. However, indications exist of alternative pathways for particles to enter cells, as studies have reported intracellular NPs of diverse materials lacking surrounding membranes [23–24]. Therefore, the occurrence of NP diffusion through pores in the membrane as well as passive uptake by van der Waals or steric interactions (subsumed as adhesive interactions) [25] cannot be excluded.

The majority of uptake studies were conducted with one defined NP type only. However, since humans are more likely to be exposed to multiple NP types present in many commercially available consumer products such as cosmetics or food, an important question is whether the uptake mechanisms for one particle type differ between single- and co-exposure of different NP types. However, only a few studies have investigated the biological effects of a combined exposure. One example was the co-exposure of epithelial A549 lung cells in cultures to carbon black and iron oxide NPs. It was reported that exposing cells simultaneously to these NPs caused a synergistic oxidative effect, which was significantly greater than the effects of exposure to each individual NP [26]. Moreover, little is known about how co-exposure of different NPs affects cellular uptake (e.g., whether it enhances or even inhibits selective NP uptake), which is –particularly in nanomedicine– highly relevant to the understanding of their behaviour in a complex colloidal system, such as the vascular system.

The aim of this work was to study the combined effect of two model NPs on cellular uptake, that is gold (AuNPs) and iron oxide NPs (FeO*_x_*NPs) stabilized with the same polymer shell and incorporated fluorophores. The NPs can be distinguished by analytical methods to assess their cellular uptake mechanisms and intracellular distribution after uptake at the single-cell level. Both NP types were provided to the cells both as single exposure, or combined as co-exposure. The NP behaviour in complete-serum-containing cell culture media was monitored by dynamic light scattering and the dose deposited onto the cell surface was determined by a modelling approach. The murine macrophage cell line J774A.1 was then used to study uptake and intracellular fate by means of laser scanning microscopy (LSM), environmental scanning electron microscopy (ESEM), transmission electron microscopy (TEM), while quantification of gold and iron oxide was performed by inductively-coupled plasma optical emission spectrometry (ICP-OES). The endocytotic route was determined by the use of specific inhibitors.

## Results

### Particle characterisation in cell culture medium and delivery onto the cell surface

**Figure 1:**
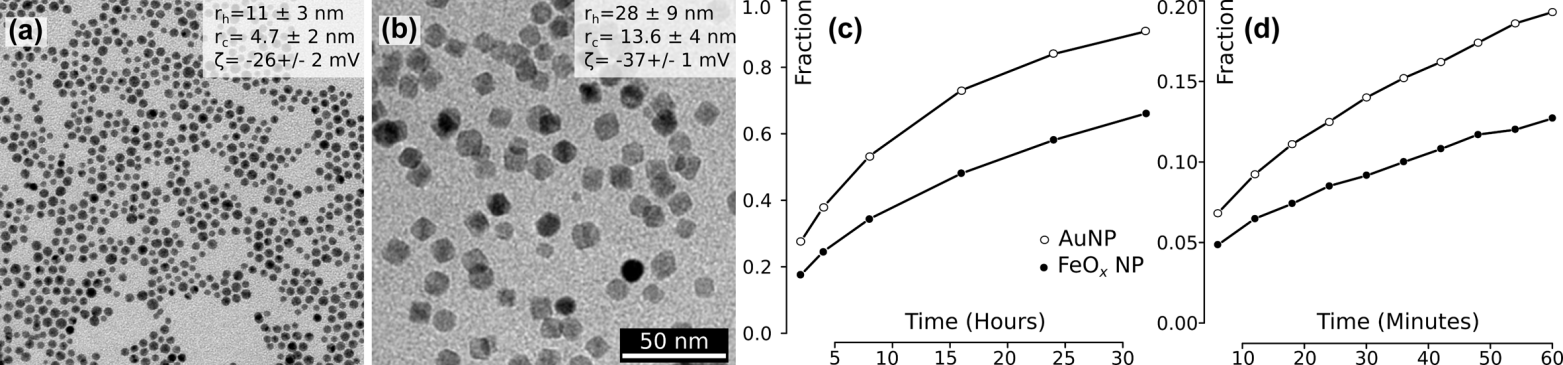
Characterization of the NPs used and their calculated deposition on the cellular monolayer. Transmission electron micrographs of (a) AuNPs and (b) FeO*_x_*NP; both images were recorded at the same magnification. Theoretical deposition of both NP types onto the cellular monolayer over 24 h (c) and over the first 60 min (d).

A general summary about the physicochemical properties of polymer-coated AuNPs and FeO*_x_*NPs can be found in a recent overview article, in which also all methods are discussed in detail [27]. The measured particle size revealed an average hydrodynamic diameter (*d*_h_) of approximately 11 ± 3 nm for AuNPs and 28 ± 9 nm for FeO*_x_*NPs, and an average core diameter *d*_c_ of 4.7 ± 2 nm for AuNPs and 13.6 ± 4 nm for FeO*_x_*NPs. AuNP was labeled with DY505 and FeO*_x_*NP with DY615; both particle types had negative surface charges of −26 mV and −37 mV, respectively (Figure 1). Depolarized dynamic light scattering measurements show that all particles analysed remained stable in both single and co-exposure experiments over 24 h in complete cell culture media (not shown). See Figures S1–S13 (Supporting Information File 1) for a full description of particle synthesis and characterization.

Given that the particles were polydisperse and were functionalized with a polymer, the dose delivered near the cell was estimated by approaches reported elsewhere, taking into account the geometrical parameters of the cell culture wells, such as overall fluid volume and well diameter [28–29]. The colloidal stability was preserved in both tests, and aggregation did not occur. Therefore, given the effective mass densities and hydrodynamic diameters, the transport-to-cell process was dominated by diffusion, while sedimentation was negligible. Our model assumes that upon reaching the cell, the NPs adhere to it, and the delivered dose is expressed as the fraction of the administered dose (Figure 1). AuNPs deposited faster than FeO*_x_*NPs, and hence a higher fraction of AuNPs was in contact with the cells throughout the experiment. After 5 min, more than 5% of the administered dose arrived at the cellular level, increasing steadily to almost 20% after 1 h. After 24 h, about 80% of the AuNPs could be considered to be in the vicinity of the cells. The deposited fraction of FeO*_x_*NPs lagged slightly behind with 2% of the particles deposited at the bottom of the culture after 5 min, more than 10% after 1 h and 50% after 24 h.

**Figure 2:**
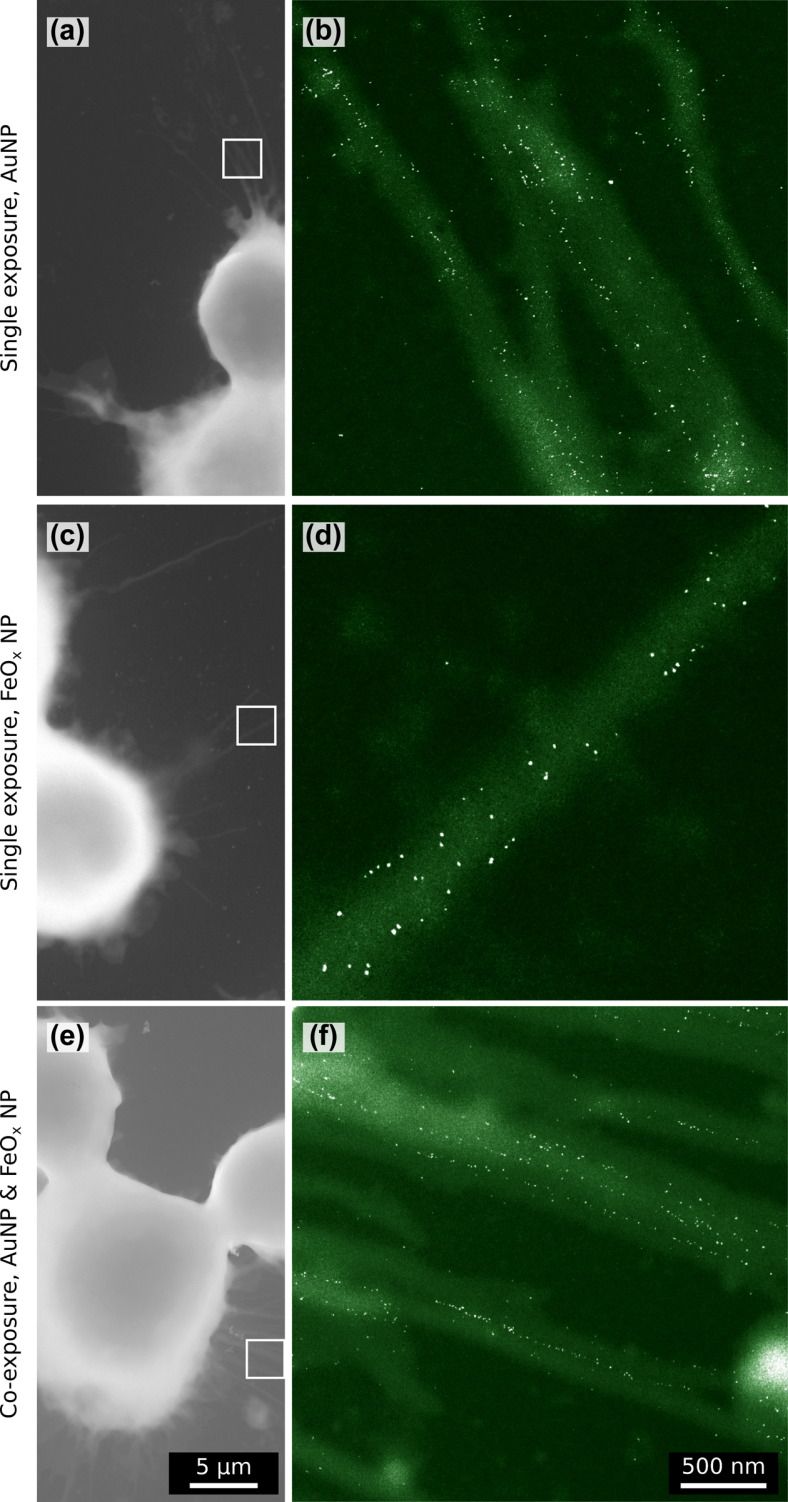
Environmental scanning electron microscopy of J774A.1 macrophages 5 min after NP exposure. Both species of NPs appear much brighter than the cellular material in the dark-field transmitted signal. Top: AuNPs in single exposure in (a) overview and at (b) high resolution. Middle: FeO*_X_*NPs in single exposure in (c) overview and at (d) high resolution. Bottom: Co-exposure of AuNPs and FeO*_X_*NPs in (e) overview and at (f) high resolution. At higher magnification (right image panel) the cell filopodia are shown in green and the particles in white.

J774A.1 mouse macrophages were exposed either to a single exposure of either particle type, or a co-exposure, and were subsequently studied using ESEM. Interestingly, both NP types were already visible at the macrophage filopodia after this short incubation time (Figure 2) and appeared as single particles (clearly excluding larger agglomerates). On several occasions the AuNPs did not seem randomly distributed but were apparently guided along linear tracks, parallel along the pseudopodium. From these regions with a high organisational level, an average distance of 37 ± 13 nm between AuNPs could be calculated (Figure S14, Supporting Information File 1). Such organisation was not observed for FeO*_x_*NPs, which appeared to be randomly dispersed on the cell surface.

### Interaction of particles with macrophages

First, it was studied if the cells indeed react on NPs in their environment and if so, on which time scale. None of the exposure conditions impaired the cell viability of J774A.1 after 24 h as shown by the LDH and trypan blue exclusion assays (Figure S15 and Figure S16, Supporting Information File 1).

**Figure 3:**
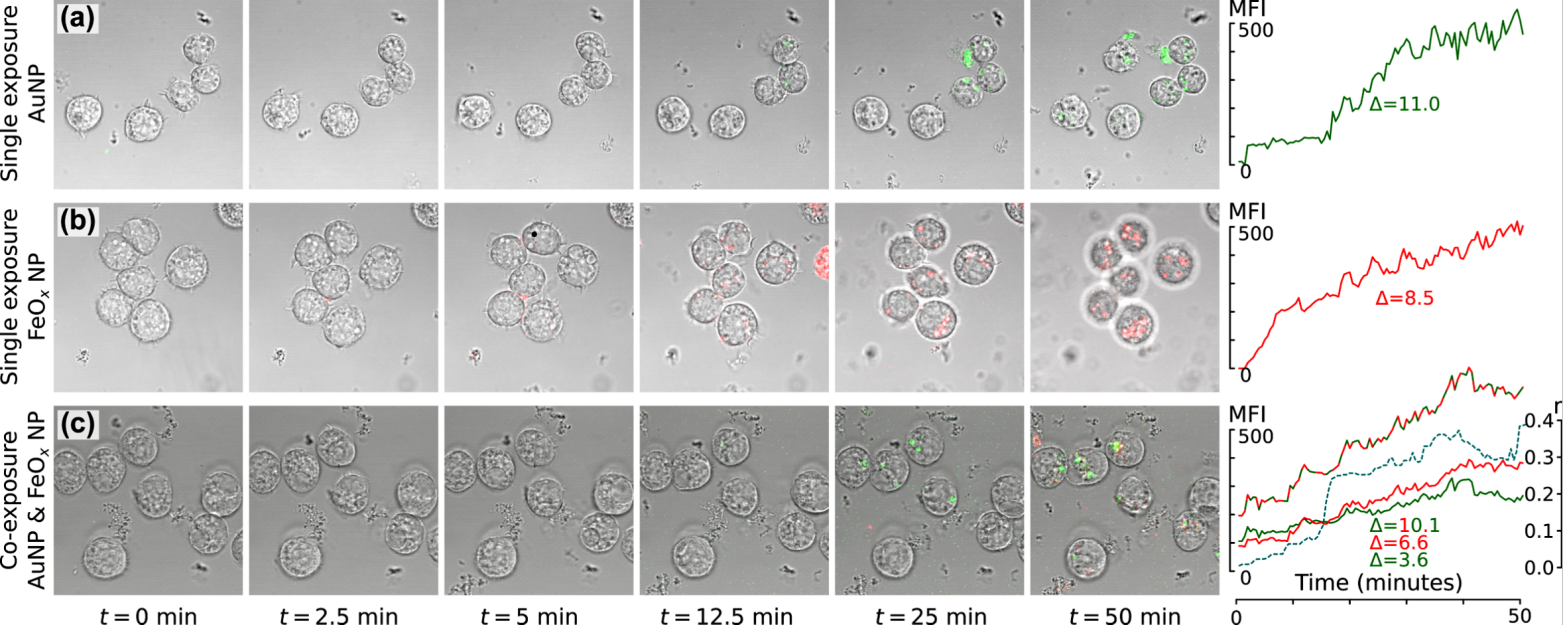
Live cell imaging via laser scanning microscopy over 50 min after the onset of NP exposure. AuNPs are tagged with a green fluorochrome, FeO*_x_*NPs with a red fluorochrome. (a) Green fluorescence accumulates in the cells. The intracellular mean fluorescence intensity (MFI) increases over time with a slope Δ of 11.0. (b) Red fluorescence of the FeO*_x_*NPs accumulates in the cells according to a slope similar to that seen for AuNPs. Note that the cells slightly move out of focus over time (*t* = 50 min). (c) The accumulation in co-exposure of either NP type is lower than in single exposure (green line for AuNP and the red line for FeO*_x_*NPs) but when combined the slope (dashed red-green) reaches the same extent as observed for the single exposures. AuNPs and FeO*_x_*NPs increasingly colocalize (yellow pixels) as evidenced by the increasing Pearson’s colocalisation coefficient, *r*. Nonetheless, after 50 min, a value of *r* = 1 was not reached as separate intracellular red and green regions remain.

Live-cell LSM data revealed that both particle types appeared intracellularly within minutes (Figure 3). The uptake rate, encoded in the slope of the intracellular mean fluorescence intensity over time (Δ), was comparable between the single exposure experiments but was lower in the co-exposure experiment: roughly half for both AuNPs and FeO*_x_*NPs (Figure 3). However, the combined AuNP and FeO*_x_*NP uptake rates in co-exposure experiments again reached the levels observed in the single-exposure treatments. This suggests that a rate-limiting process was taking place. The steady increase of the Pearson's colocalisation coefficient (*r*) of AuNPs and FeO*_x_*NPs (intracellularly measured, blue dotted line) over time is an indication that both particle types can end up in the same cellular compartments: However the Pearson’s coefficient never reaches the maximum value of one (perfect colocalization) and intracellular regions that were either marked for AuNPs or FeO*_x_*NPs were always observed.

**Figure 4:**
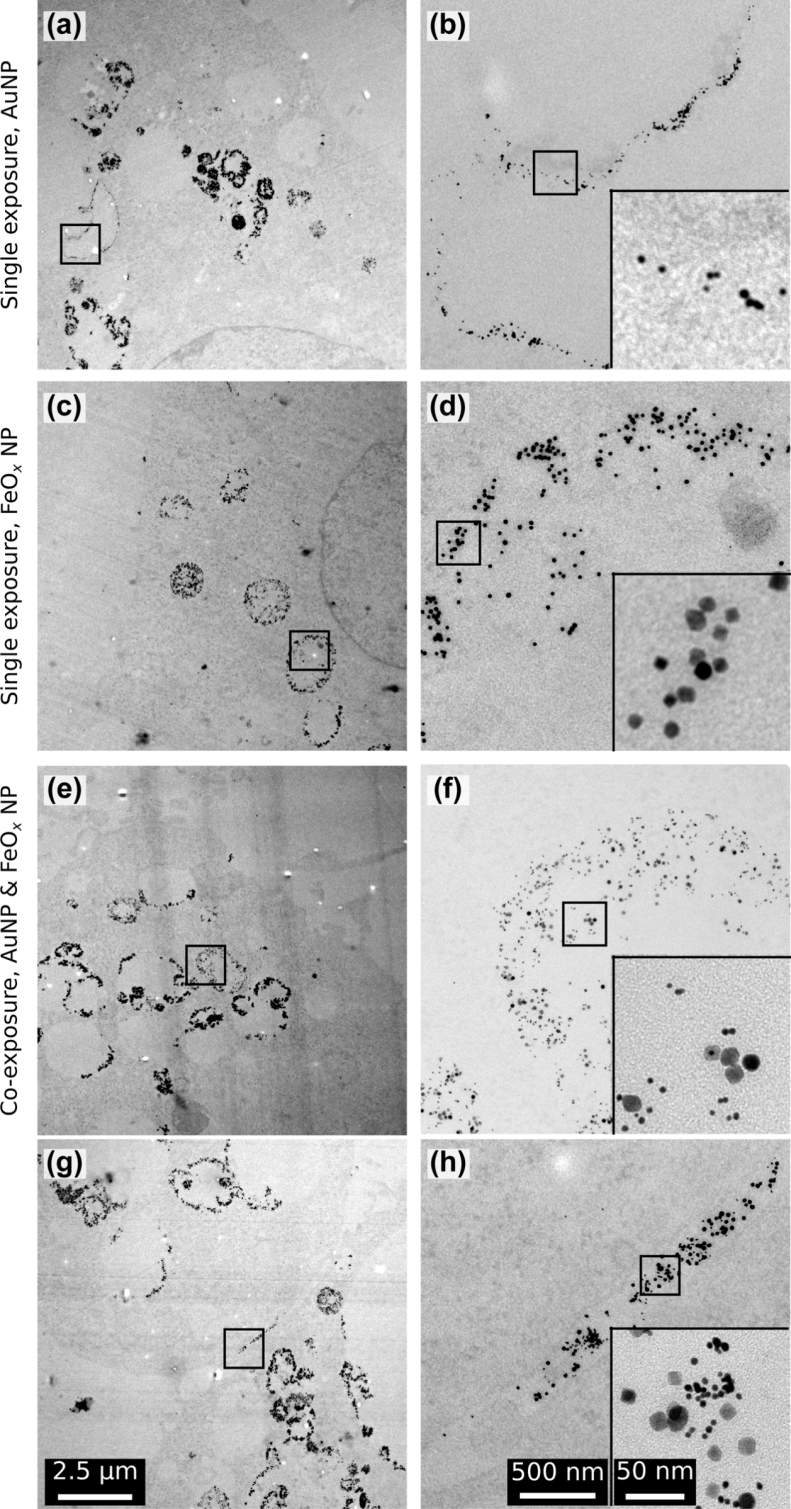
The intracellular localisation of the NPs 24 h after exposure. (a) Overview showing a part of a cell after exposure only to AuNPs with a number of AuNP-containing vesicles. (b) The higher magnification of the square in (a) reveals that particles are inside the vesicle and appearing both as single entities and aggregates (inset). (c) Overview showing a part of a cell after exposure to only FeO*_x_*NPs with FeO*_x_*NPs-filled vesicles. (d) The particles appear loosely aggregated (inset) and are contained within the border of the vesicle. (e) Overview showing a part of a cell after co-exposure with AuNPs and FeO*_x_*NPs. (f) Both particle types co-appear inside vesicles, either as single particles or small aggregates (inset). (g) A small extracellular crevasse between two cells co-exposed with AuNPs and FeO*_x_*NPs is filled with both particle types. (h) Both particle types are mixed extracellularly, in single or aggregated status (inset). All overviews, higher magnifications and insets have a scale bar of 2.5µm, 500 nm and 50 nm, respectively.

Colocalisation of both particle types in intracellular compartments 24 h after exposure was confirmed by TEM (Figure 4). Intracellular particles were found only in vesicles and in the co-exposure experiments, both particle types were observed in the same vesicle. In the immediate extracellular vicinity of the cell membrane events of colocalizing AuNPs and FeO*_x_*NPs were also found (Figure 4).

### Quantification of particle uptake

**Figure 5:**
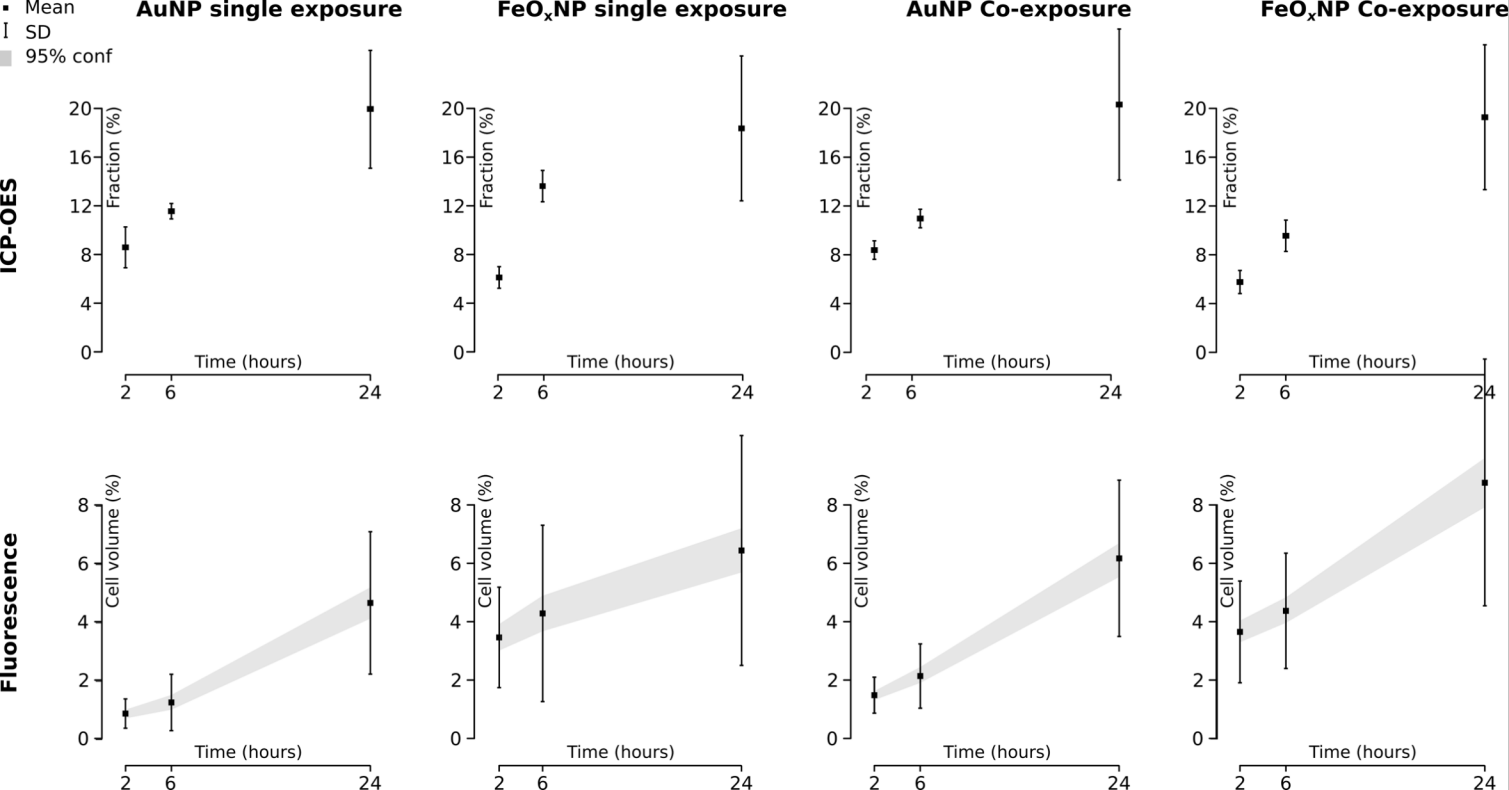
Quantification of uptake by ICP-OES and LSM combined with digital analysis. ICP-OES measurements reveal an increasing trend of NP uptake, independent of NP type and exposure mode. The LSM data supports the ICP-OES data, i.e., linear uptake trends were found for both NP types and exposure modes. Only for single exposure with FeO*_x_*NPs could a slight derivation of linearity be observed. This effect was seen in both methodologies. The data show mean ± one standard deviation. The grey regions give the 95% confidence interval (ICP-OES: *n* = 6 cultures, LSM 10 *z*-tacks per exposure type and particle type, yielding minimum 61 cells and maximum 104 cells, hence 60 < *n* < 105).

The quantification of the intracellular fraction of elemental Au or Fe (Figure 5, top) by ICP-OES revealed a continuous uptake of both particle types in all exposure modes over the time range of 24 h. The cleaned fraction of the medium was always lower than the delivery of the model (see Figure 1) predicted, again hinting at a rate-limited uptake process. Neither particle type nor exposure setting influenced the uptake fraction after 24 h, which was about 18% in all cases.

These positive correlations between uptake and time are confirmed by the LSM data. A continuous increase in the number of voxels associated with a fluorescence signal (i.e., the uptake load) within the investigated time window was found, independent of particle type or exposure mode (Figure 5). The NP load after 24 h was significantly higher (*p* < 0.05) than that after 2 h, for all NP types and exposure modes. The co-exposure condition appeared to increase the uptake rate, resulting in higher NP loads of both types after 24 h. After 2 h in the co-exposure experiments, 66% more voxels can be attributed to AuNP than in single-exposure experiments. This effect lasted the entire experiment, with still 31% more AuNP after 24 h in co-exposure experiments. A similar effect is seen in the FeO*_x_*NP uptake: 37% more voxels were associated with these particles after 24 h in co-exposure experiments, compared to single-exposure experiments after the same time.

**Figure 6:**
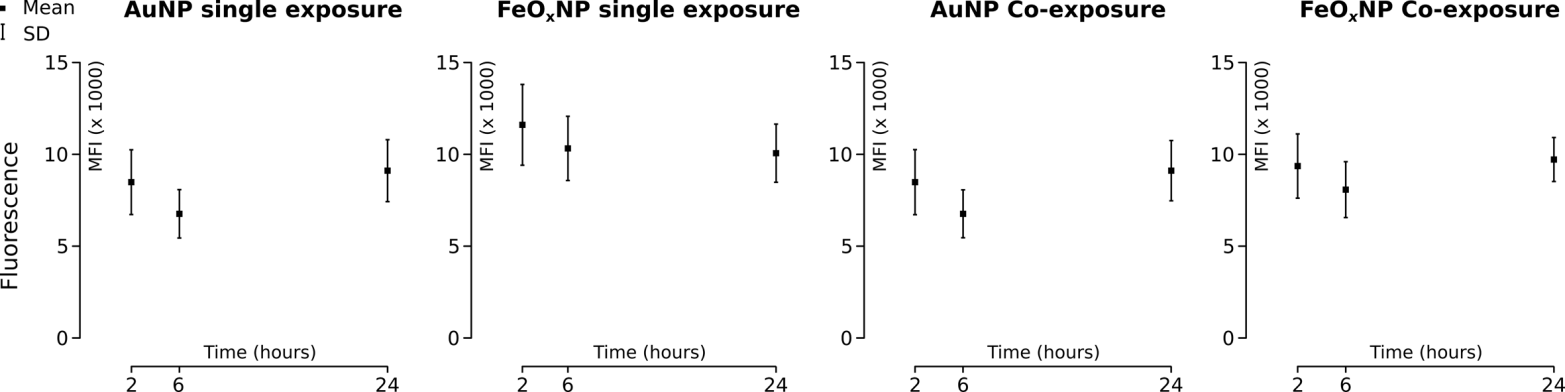
Mean fluorescence intensity of the intracellular pixels over time. The intensity of the pixels associated with NPs remains statistically stable over the entire experiment. The data show mean ± one standard deviation. *n* = 10 *z*-stacks per exposure type and particle type, yielding a minimum of 61 cells and a maximum of 104 cells, hence 60 < *n* < 105).

The intracellular mean fluorescence intensity did not change over the course of the experiment: no significant difference (*p* < 0.05) in intracellular mean fluorescent intensity between 2 h, 6 h and 24 h for any of the NP types nor for any exposure mode was found (Figure 6). A number of convoluted factors influenced the average fluorescence in a cell: The cell cycle influences the uptake rate and after mitosis (population doubling time: about 17 h) the NP load is split between the two daughter cells [30]. Furthermore, the uptake of NPs requires the production of cellular components, such as membrane to supply for vesicles. It can therefore be expected that the cell volume changes, which will also entail changes in average fluorescence. These factors may explain the lack of fluorescence change over time, or the macrophages may simply not accumulate particles, or at least not within this time range.

### Intracellular fate of nanoparticles

After uptake, the question of intracellular fate, in particular, the question of potential NP colocalization inside the cells in relation to known cellular compartments was raised. This applied in particular to the compartments involved in uptake pathways, such as lysosomes.

**Figure 7:**
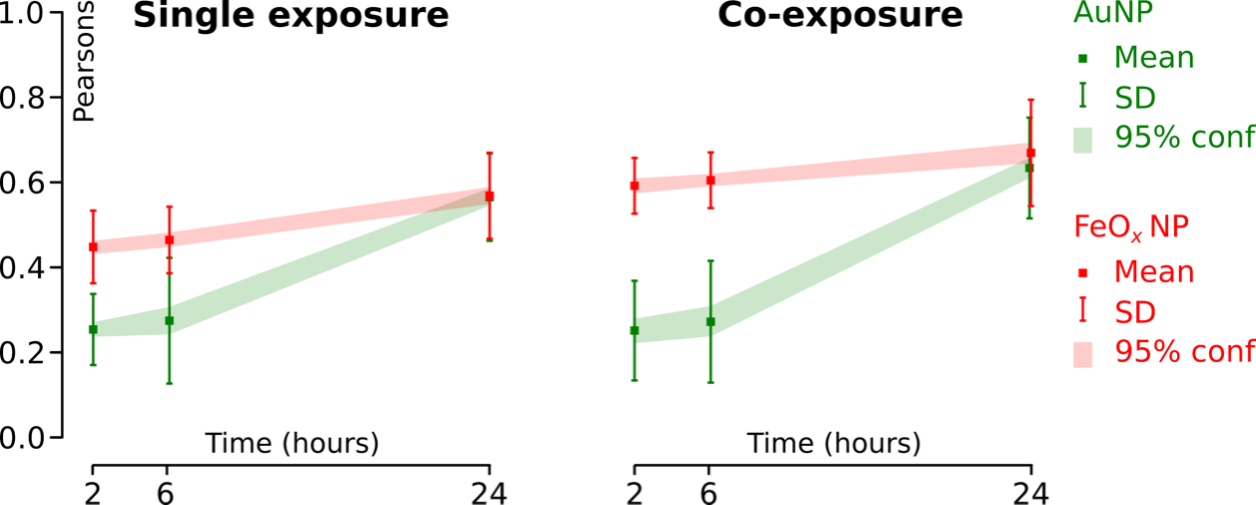
Time-dependent colocalisation of AuNPs and FeO*_x_*NPs with lysosomal markers. The Pearson’s colocalisation coefficient increases over the time span between 2 and 24 h, but is much higher for AuNPs than for FeO*_x_*NPs. This observation can be attributed to the already high colocalization of FeO*_x_*NP with lysosomal markers after 2 h. The data show mean ± one standard deviation. The green and red regions link 95% confidence intervals for AuNPs and FeO*_x_*NPs, respectively (*n* = 25 *z*-stacks).

A significant difference in behaviour between AuNPs and FeO*_x_*NPs in relation to the localisation in lysosomes was observed (Figure 7). While a strong overlap between FeO*_x_*NPs and lysosomal markers was observed even after only 2 h, which resulted in a high Pearson’s colocalisation coefficient (0.45 in single-exposure experiments, 0.56 in co-exposure experiments), this was not the case for AuNPs. A weak positive colocalisation with lysosomal markers after 2 h was reflected by the low Pearson’s colocalisation coefficient (0.25 in single-exposure experiments, 0.21 in co-exposure experiments). Colocalisation between FeO*_x_*NPs and lysosomes increased only marginally during the last 22 h of the experiment. Again a different situation was observed for AuNPs: A steep increase in colocalisation with lysosomes was observed between 6 and 26 h after exposure (*r* = 0.60 and 0.57 for single- and co-exposure experiments, respectively). After 24 h, both particle types colocalize to approximately the same extend with lysosomal markers, independent of the exposure mode.

**Figure 8:**
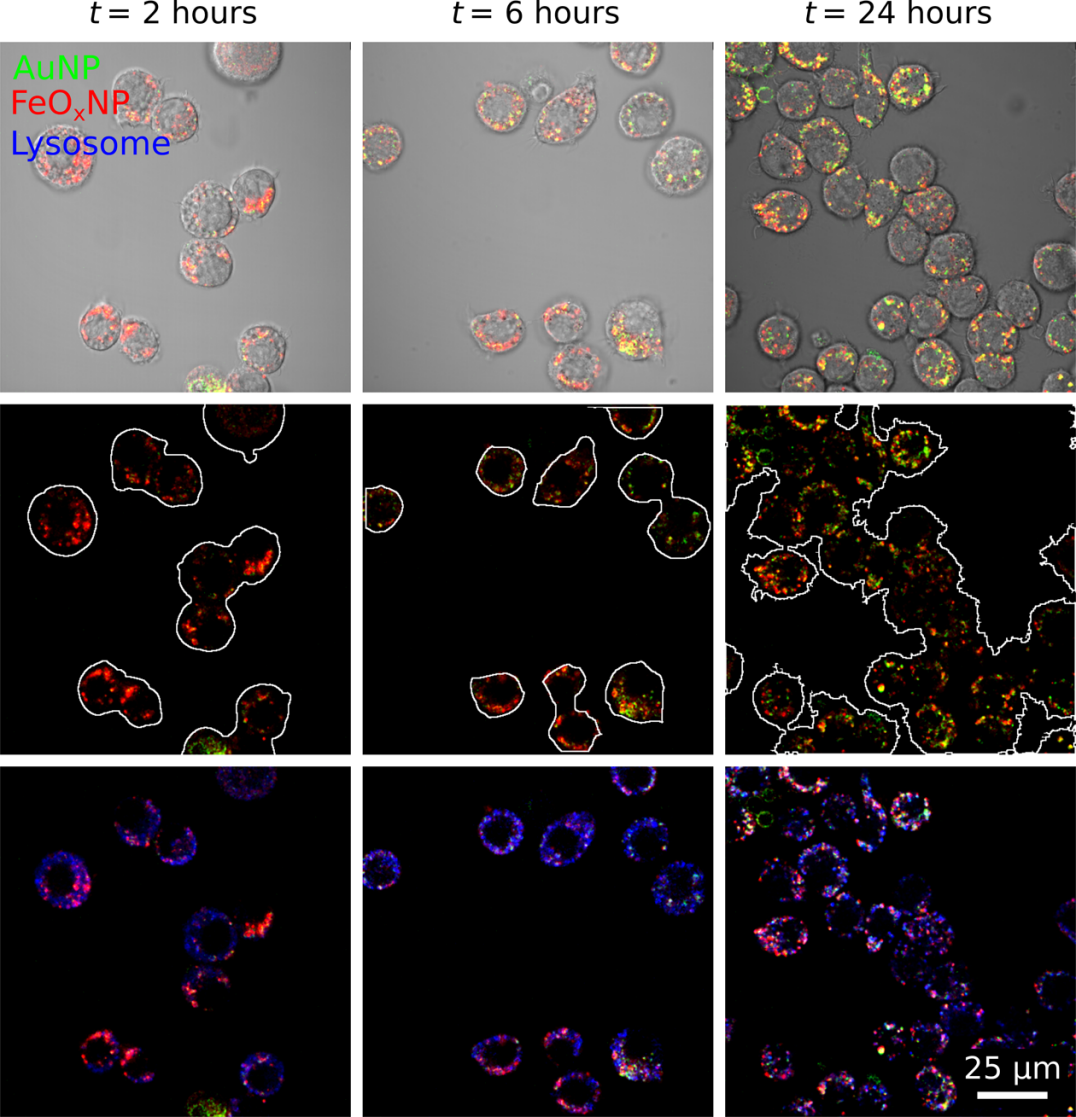
Colocalisation of AuNPs and FeO*_x_*NPs with the lysosomal marker. The interplay of AuNPs, FeO*_x_*NPs and lysosome hints at the complexity of the uptake mechanisms involved. The two NP types colocalize (yellow) but not completely: Exclusively green (AuNP) or red areas (FeO*_x_*NP) remain, even after 24 h of exposure. Meanwhile, the overlap with lysosomal markers is undeniable, but again, not complete. Additionally, it is clear that, on average, the FeO*_x_*NPs (red channel) arrive in the lysosomes first. While a considerable FeO*_x_*NP signal can be detected after 2 h, AuNPs are still nearly undetectable. After 24 h, the situation has levelled off. Top row: Bright-field image with the two NP fluorosence signals overlaid. Middle row: Outlined cells, based on the bright-field image, overlaid with the two NP fluorescence channels. Bottom row: lysosomal marker overlaid with the two fluorescence channels.

Additionally, in co-exposure experiments, AuNPs and FeO*_x_*NPs increasingly colocalise, confirming the observed onset of this effect as previously shown in the 2D LSM data of Figure 3. However, the Pearson’s colocalisation coefficient levels off at around 0.55 after 24 h (Figure 4), and in addition to coinciding regions, there remain intracellular regions that were exclusively attributed to the fluorescence signal of either the AuNPs or FeO*_x_*NPs (Figure 8).

The colocalisation of both NP types in other cell organelles was also investigated and no signal overlap between NP events and the Golgi apparatus, the mitochondria or the nucleus was found after 24 h (data not shown).

### Uptake mechanisms

Inhibitors were used to investigate the endocytotic uptake mechanisms of the two NP types. All inhibitors have previously been optimized and tested in J774A.1, showing that phagocytosis, micropinocytosis and clathrin-mediated endocytosis are the main uptake mechanisms in this cell type [31].

**Figure 9:**
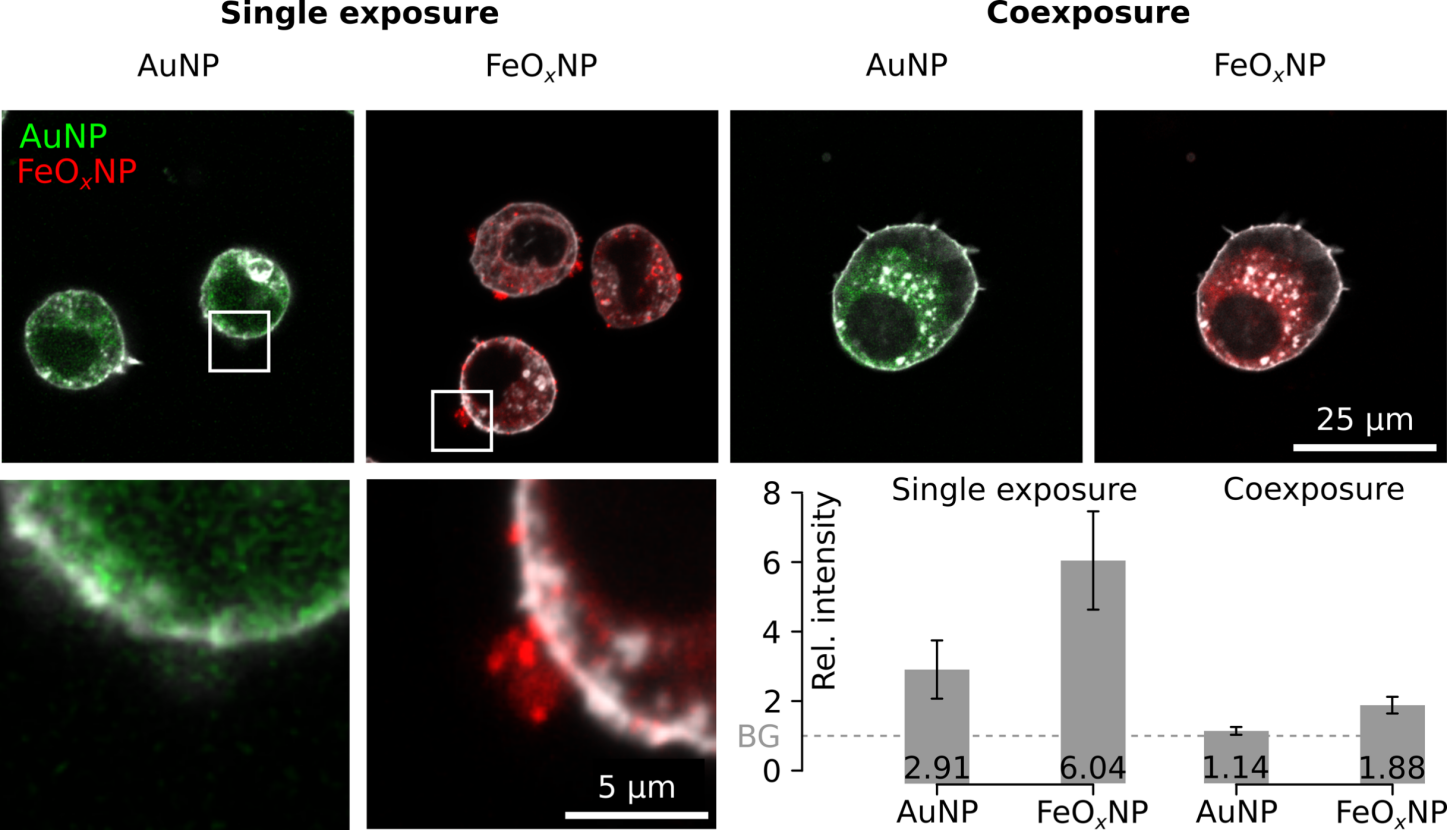
Effect of MDC treatment on the uptake of AuNPs and FeO*_x_*NPs. The uptake is partially blocked in the single-exposure experiments. Aggregates were seen in the vicinity of the cell (larger magnification, with resolution loss at bottom left). This situation is rectified (for AuNPs) or almost rectified (for FeO*_x_*NPs) in the co-exposure experiments. The quantification is based on the average fluorescence in an extracellular area 120% the size of the cell (“the vicinity”). The graph report values relative to the average background (BG), as retrieved from the area marked by the dotted line in the figures (*n* = 5).

The treatment of the cells with cytochalasin D (phagocytosis and micropinocytosis inhibitor) had no effect and both NP types could still be localized intracellularly (data not shown). The monodansylcadaverine (MDC, clathrin-mediated endocytosis inhibitor) impaired the uptake of AuNPs, and FeO*_x_*NPs: Aggregations on the outer cell membrane were observed (Figure 9). In the single-exposure experiments, the average fluorescence in the vicinity of the cell, set at the 20% surrounding rim outside the cell (Figure S18, Supporting Information File 1), was significantly higher (*p* < 0.05) than the average background values extracted from a region 15 µm away from the cell and denoted by the dashed circle (Figure 9). The average AuNP fluorescence was almost 3-fold increased and the average FeO*_x_*NP fluorescence more than 6-fold. This situation was completely rectified in the co-exposure experiments for AuNPs. The fluorescence in the vicinity of the cell did not differ from the background (1.14 times background, no significant difference). In the case of FeO*_x_*NPs, the average fluorescence in the vicinity of the cell (1.88 times background) was significantly lower (*p* < 0.05) than in single-exposure experiments, but still higher than the background levels. In general, the co-exposure setting reduced NP fluorescence in the 20% vicinity of the cell to about one third, independent of the particle type.

## Discussion

J774A.1 macrophages react differently to AuNPs and FeO*_x_*NPs, even if these particles are administered simultaneously and at a similar load. Both types of NPs have a similar polymeric surface coating. However, the core materials (gold versus iron oxide) and their overall size differ and can both influence the interaction with cells. The core material can, for example, cause catalytic effects, which have been reported to a higher extend in the case of gold than for iron oxide [32]. This difference has also been reported to influence pro-inflammatory response of cells upon exposure of cells to NPs [33]. Also, the overall size is known to affect NP uptake by cells [34]. Thus, while two parameters were intentionally varied at the same time in order to obtain different uptake pathways, which of the two parameters and to what extent was responsible to cause the respective pathways is unknown, and beyond the scope of this study.

The uptake of the particles involves clathrin, since their uptake was partially blocked by the clathrin inhibitor MDC. Inhibition of this pathway was also reported for the Fe-binding protein transferrin, whereas the uptake of non-biological (nano)particles such as polystyrene beads was not blocked [35]. However, the pathway was not exclusively clathrin-mediated because evidence of uptake is observed intracellularly after MDC treatment, suggesting that at least one additional uptake mechanism is active. Co-exposure of FeO*_x_*NPs and AuNPs in the presence of the clathrin-mediated endocytosis inhibitor MDC mediated a recovery from the uptake inhibition, again suggesting that more than one pathway is involved. Therefore, the co-exposure of NPs to a culture of macrophages with an inhibited clathrin-mediated endocytosis pathway may restore the uptake until it is indiscernible from a not-inhibited, healthy culture. Without inhibition, the particles are internalized faster in co-exposure experiments than in each single-exposure experiment. This may initially appear paradoxical can be easily explained by the activation of two parallel uptake pathways.

After internalization, the fluorescent signals from AuNPs and FeO*_x_*NPs never completely colocalise, which may again be a reflection of different uptake mechanisms. Uptake occurs in a “first come, first served” manner, as suggested by the uptake slopes seen in the live-cell imaging and supported by the ICP data. Also the TEM data did not find evidence for particle-specific sorting before the uptake.

A distinct type of organelles, namely caveosomes [36], has been suggested as target for caveolae-mediated endocytosis. The nature of this organelle is still disputed but it may act as a relay station for caviculae trafficking, whereas clathrin-mediated endocytosis delivers directly into the early endosomal/lysosomal system [37]. The caveolin system interconnects with the endosomal system at two points: The first is a pathway from the plasma membrane to the early endosomes where endocytosed cavicles briefly encounter (kiss and run) early endosomes and may exchange some cargo and the second is to the late endosomes/lysosomes (observed when cells were starved or the lysosomal pH value dissipated) [37]. These putative pathways explain our observations satisfactorily. The observed differences in delivery time to the endosomal system between AuNPs and FeO*_x_*NPs can be attributed to the AuNPs being held up in the caviculae trafficking system, which does not colocalize with lysosomal markers. FeO*_x_*NPs, on the other hand, are thought to be delivered directly to the endosomal/lysosomal pathway by the clathrin-dependent endocytosis uptake and quickly colocalize with the lysosomal markers. The occurrence of AuNPs in the lysosomes does eventually increase, until the difference to FeO*_x_*NPs is minimal (observed after 24 h). A shuttle between cavicles and late endosomes has been described [38], but it requires starving cells or lysosomal perturbations. Since starving was not induced, the origin of this observation may again be found in the convoluted uptake mechanisms of AuNPs. Since we found no evidence that a sorting step prior to uptake is taking place, it is conceivable that AuNPs also enter the cell by unbiased endocytosis. The TEM data qualitatively supports this hypothesis since both particle types were seen extracellularly in close company (few nanometres apart) and vesicular structures contain a mixture of both AuNPs and FeO*_x_*NPs after 24 h.

Within 5 min after exposure AuNPs were seen in ESEM. Associated with filopodia, the AuNPs exhibit remarkable organizational behaviour. Some AuNPs were arranged along lines, parallel to the longitudinal axis of the filopodia. Since a similar organization was not observed for the FeO*_x_*NPs, it must be concluded that an active process directed the uptake of AuNPs. A similar observation was made with 1 µm polystyrene beads, which were observed to be transported extracellularly along the filopodia of the same macrophageal cell line [39]. Furthermore, it was shown that exosomes moved along the extracellular side of filopodia prior to internalization [40]. The transport along filopodia has been suggested to be mediated by the underlying actin–myosin cytoskeleton [41]. The distance between linearly arranged AuNPs averaged 37 nm, which matches the repetition length reported for structures of actin filament bundles and actin branches [42]. It is conceivable that AuNPs were transported at the extracellular side of the plasma membrane towards an entry point for endocytosis. This could also explain the delayed uptake of AuNPs in comparison with FeO*_x_*NPs. However, it should be noted that the presence of an intracellular transport system for the AuNPs cannot be ruled out completely.

## Conclusion

Most studies on NPs focus on exposure to a single analyte. The existing literature on joint toxicity of NPs and co-existing contaminants is rather limited but beginning to develop rapidly [43]. We contribute to this literature by examining the co-exposure of AuNPs and FeO*_x_*NPs. We conclude that at least two uptake pathways are involved and that there is crosstalk between the pathways. The co-exposure setting was able to rescue the cell from of clathrin-mediated pathway inhibitor, hinting at synergetic effects between the two particle types and their activated uptake pathways. The co-exposure mode increased the uptake, independent of NP type, but not the time to appear in lysosomes. These finding have relevance for toxicological studies: Co-exposure acts as an uptake accelerant and may be beneficial in biomedical settings if the goal is to maximize the cellular uptake, e.g., for the delivery of a pharmaceutical agents. However, co-exposure may cause negative effects in the case of risk assessment of occupational settings. In any rate, the demonstration of interactions between different particle types at the cellular level reveals that synergetic effects, either positive or negative, must be considered for nanotechnology and nanomedicine in particular to develop to its full potential.

## Experimental

### Materials

HPLC grade acetone, ethanol, and deionized water, phosphate-buffered saline (PBS) at a 10× solution, electron microscopy grade glutaraldehyde (GA) 25% solution, D-saccharose, glycine, biotin-free and molecular biology grade albumin fraction V (BSA), and sodium cacodylate trihydrate were obtained from Carl Roth GmbH + Co. KG, Karlsruhe, Germany. 0.01% poly(L-lysine) solution (PLL, *M*_r_ = 150,000–300,000, sterile-filtered, and cell-culture tested) was purchased from Sigma-Aldrich Chemie Gmbh, Munich, Germany. Sample support microchips with a central silicon nitride (SiN) membrane window of a dimension of 150 × 400 μm and a thickness of 50 nm were custom-made by DENSsolutions B.V. (Delft, Netherlands). The chips had outer dimensions of 2.00 × 2.60 × 0.30 mm^3^ fitting into the wells of a standard 96-well plate.

### Particle characterization and exposure

The details of the NP synthesis, which followed the protocols published in [44–45], and characterisation are provided in Supporting Information File 1 (Figures S1–S13). Please note that the synthesis protocol employed for the iron oxide NPs has been reported to yield maghemite (γ-Fe_2_O_3_), but as no experimental verification was applied to exclude formation of magnetite (Fe_3_O_4_), the NPs are referred to as FeO*_x_*NPs. Due to the short exposure and the similar surface chemistry the precise stoichiometry of the FeO*_x_*NPs is assumed to be not of major relevance in this present study. The two NP types coated with poly(isobutylene-*alt*-maleic anhydride)-*graft*-dodecylamine (PMA), i.e., AuNPs with 2% DY505 fluorophore, and FeO*_x_*NPs with 2% DY615 fluorophore integrated into the polymer shell were synthesized as previously described [44–45]. For both NPs the hydrodynamic radius (*d*_h_) was measured by dynamic light scattering (DLS) and a corresponding core diameter (*d*_c_ = 2·*r*_c_) was extracted from transmission electron micrographs. Suspensions of AuNPs and FeO*_x_*NPs with concentrations of 38.6 µg/mL and 54.8 µg/mL, respectively, were applied to a cultured mouse macrophage system (see below). The colloidal stability of the particles in complete cell culture media containing 10% fetal bovine serum (FBS) and 1% L-glutamine was measured by depolarized dynamic light scattering (DDLS).

### Calculation of administered NP doses

The total mass of NPs in a solution of volume of *V* is given by:

[1]
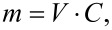


where *C* is the mass-based concentration of the particle suspension. Equation 1 can be written as:

[2]
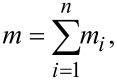


where *n* is the total number of particles and *m_i_* is the mass of the *i*-th particle. The particles exhibit some polydispersity as both the core and the hydrodynamic radius show a finite dispersion. Equation 2 can be expressed by the volume (V*_i_*_,core_, V*_i_*_,shell_) and mass density (ρ) of the (polymer) shell and core of the particles:

[3]
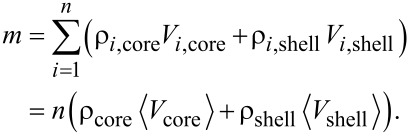


The brackets indicate the average value:

[4]
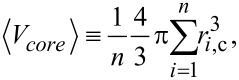


[5]
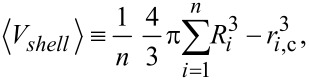


where *r_i_*_,c_ and *R_i_* are, respectively, the radius of the core and hydrodynamic radius of the *i*-th particle. Finally, the overall number of particles is

[6]
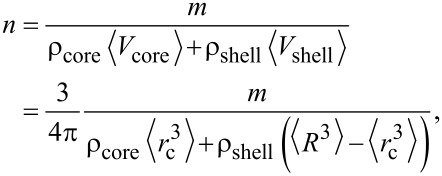


where

[7]
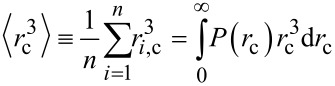


is the third raw moment of the particle size distribution *P*(*r*_c_). We describe both *r*_c_ and *R* by a normal distribution (μ being the mean and σ being the standard deviation) whose third raw moment is given by

[8]
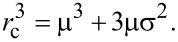


Since ICP-OES measures only the elemental core and not the polymer shell, Equation 6 is adapted as follows:

[9]
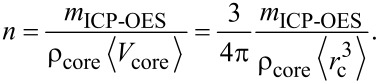


The mass density of the gold was taken as 19.2 g/cm^3^, and 5.24 g/cm^3^ was taken for the mass density of the iron oxide.

### Cell cultures

Mouse macrophage cells (J774A.1 cell line) received from the American tissue Type Culture Collection were cultured in RPMI 1640 with HEPES (Gibco, Luzern, Switzerland) completed with 10% fetal bovine serum (FBS, heat inactivated, PAA Laboratories, Austria), 1% L-glutamine (Gibco, Luzern, Switzerland) and 1% penicillin/streptomycin (Gibco, Luzern, Switzerland) and were kept at 37 °C and 5% CO_2_. The cells were seeded at a density of 25 × 10^4^ cells/mL in BD Falcon^TM^ four-chamber polystyrene vessels for the experiments, using tissue culture treated glass slides with a growth area of 1.7 cm^2^ (Milian, Geneva, Switzerland) and grown for one day.

### Endocytosis inhibitor experiments

Inhibition of clathrin-mediated endocytosis was performed with monodansylcadaverine (1 h, 250 µM; dansylcadaverine, D4008, Sigma-Aldrich). Cytochalasin D (1 h, 4 µM; C8273, Sigma-Aldrich) was used to inhibit phagocytosis and micropinocytosis.

### Cell fixation and labelling for laser scanning microscopy (LSM)

To label the proteins involved in endocytotic uptake, the cells were fixed with 4% paraformaldehyde (PFA, Sigma-Aldrich, Switzerland) in phosphate-buffered saline (PBS) for 15 min at room temperature (RT), washed once with PBS and permeabilized for 15 min with 0.2% Triton X. Antibodies against clathrin heavy chain and flotillin-1 (both labelled with Alexa Fluor 488 and Cy-3; Antibodies-Online GmbH, Aachen, Germany) were used at a final dilution of 1:20 in 1× phosphate-buffered saline (PBS). After 1 h of staining (in the dark at RT) and three washing cycles with 1× PBS the cells were mounted using Glycergel mounting media (C0563, Dako, Baar, Switzerland).

For live-cell imaging, cells were seeded in a Lab-Tek^TM^ II chambered cover glass four-well chamber (1.5 German cover glass system, NC-155382, Nunc, Milian, Geneva, Switzerland) and kept at 37 °C and 5% CO_2_. Lysotracker to stain lysosomes, Mitotracker to stain mitochondria and Nunc Blue to stain the nucleus (Molecular Probes) were added to the cells for 1 h, after which the cells were washed once with PSB to remove any leftover dye. The samples were immediately examined after the labelling.

### Laser scanning microscopy and data restoration

Image acquisition was performed with an inverted Zeiss LSM 710 Meta apparatus (Axio Observer.Z1, Zeiss, Switzerland) equipped with 405 nm diode, and 488, 561 and 633 nm laser lines. The analysis was performed using a 63×/NA 1.4 immersion oil lens.

### Image processing routines

Analytical data were retrieved from the laser scanning micrographs (stacks of bright field and fluorescence channels) by means of dedicated ImageJ macro scripts (see Supporting Information File 1). In short, the raw bright field image was used to retrieve the outline of the cell using a combination of variance and median filters. This outline was used as a mask on the fluorescence images (Figure S17, Supporting Information File 1). Individual pixel information was retrieved by a scanning routine over the dataset, including only elements that were marked as belonging to the cell by the bright field mask. The data for the Pearson’s colocalisation coefficient, notably the fluorescence intensity value at each pixel position *x_i_* and *y_i_*, was obtained in a similar way using

[10]
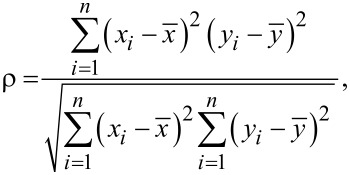


with 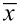 and 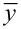 corresponding to the mean fluorescence intensity value of a total of *n* pixels of the first (*x*) and second (*y*) channel, respectively.

Alternatively, running an IsoData binarisation routine after masking yielded a subgroup of clusters of high intensity. A similar scanning routine then provided the value of each of these pixels and their total number. The full scripts can be found in Supporting Information File 1.

### Inductively coupled plasma optical emission spectroscopy (ICP-OES)

Cell uptake of gold and iron oxide NPs was measured via ICP-OES (PerkinElmer Optima 7000 DV). After NP exposure, the cells were stored at −80 °C prior to sample treatment. The thawed cells and supernatant were separately treated with a 2:1 mixture of HNO_3_ (Sigma Aldrich) and H_2_O_2_ (Reacto lab SA) (300 μL per sample), and digested for several hours. Subsequently, the samples were briefly sonicated prior to the addition of 400 μL of HCl (Honeywell AG) and digestion was performed overnight. Samples were transferred to 15 mL Falcon tubes (BD Biosciences, Switzerland) and diluted ten-fold with water. The treated samples were then measured by ICP-OES (wavelengths used: 242.795 nm for Au and 259.939 nm for Fe). A standard curve of aqueous gold and iron solutions (Fluka) was recorded to quantify the amount of intracellular gold and iron oxide. Three independent experiments were performed.

### Environmental scanning electron microscopy of whole cells in the wet state

A detailed description of the method of ESEM of whole cells in liquid can be found elsewhere [46–47]. Cells were seeded in medium at 37 °C onto silicon microchips with thin silicon nitride windows for transmission electron microscopy (TEM) [48]. The adherent cells were then incubated in the respective NP solutions for 5 min at 37 °C as described below. After exposure, washing and fixation steps, the samples were kept at 4 °C. Directly before ESEM imaging, samples were washed three times with ice-cold pure water and transferred into the electron microscope (ESEM, Quanta 400 FEG, FEI, USA). The cells were maintained in a water vapour environment at 3 °C and imaged using a scanning transmission electron microscopy (STEM) detector (described in detail below). The water layer covering the cells was carefully thinned until it was sufficiently thin to allow for the detection of single NPs in a cell with the STEM detector located under the sample. In order to easier discern cellular material from nanoparticles, the “Thorium” lookup table, available in ImageJ, was used.

### Incubation with NPs for ESEM

The AuNP and FeO*_x_*NP solutions were diluted in RPMI 1640 with 10% fetal bovine serum (heat inactivated, PAA Laboratories, Austria), 1% L-glutamine (Gibco, Luzern, Switzerland) and 1% penicillin/streptomycin (Gibco, Luzern, Switzerland) to a final concentration of 20 μg/μL. After 3–4 h of incubation to allow the cells to adhere on the microchips, the cells were rinsed once with medium, and then incubated for 5 min at 37 °C in the respective NP solutions. This was done by placing a droplet (11 μL) of the NP solution at the rim of a cuff-off lid from a PCR tube, a microchip with adherent cells was placed into the droplet such that the chip was inclined against the rim, while the cells were oriented upside down and completely immersed in the NP solution. After rinsing three times with PBS and once with 0.1 M cacodylate buffer (CB) and 0.1 M saccharose (pH 7.4), the cells were fixed with 2% GA in CB for 10 min at RT. Subsequently, the cells were rinsed once with CB, and three times with PBS, followed by incubation in 0.1 M glycine in PBS (GLY-PBS, pH 7.4) for 2 min, rinsing three times with PBS supplemented with 1% BSA, and storage in this buffer at 4 °C until electron microscopic investigation.

### Wet ESEM-STEM

An electron beam with an energy of 30 kV, a spot size of 1 nm, a probe current of 600 pA, and working distances between 6.0 and 6.4 mm were used. The stage temperature was kept at 3 °C, and the pressure was set to a value between 700 and 740 Pa, in most cases 720 Pa. These pressure and temperature settings created 100% relative humidity in the ESEM chamber as needed to ensure the constant coverage of the cells with a thin film of water. For every sample, an overview ESEM-STEM image was recorded showing the whole membrane window area with all cells. Consequently, overview images were recorded from individual cells or cell groups at a higher magnification. To discern NPs on the plasma membrane, the magnification was set to 25,000× or 50,000×, and pixel-dwell times between 30 and 100 μs were used. The image sizes were 2048 × 1768 or 1024 × 884 pixels. The electron dose for an image ranged between 81 and 376 *e*^−^/Å^2^, and was below the limit of radiation damage [49].

### Transmission electron microscopy

Fixation was carried out with 2.5% glutaraldehyde in 0.15 M cacodylate buffer. Staining occurred with osmium tetraoxide in ddH_2_O. After the staining, membranes were transferred to 0.05 M maleate buffer and dehydrated with an increased ethanol series (30% and 70%). The membranes were embedded in epon and polymerized at 60 °C. The samples were repeatedly plunged into liquid nitrogen to remove the cells from the glass slide. Ultra-thin sections of 70 nm were cut at an angle of 10° and cast on TEM single-slot grids for analysis. The micrographs were recorded on a FEI Morgagni TEM (Hillsboro, OR, USA) running at 80 kV equipped with a Olympus SIS Morada 11 megapixel camera (Münster, Germany).

### Lactate dehydrogenase assay (LDH)

One millilitre of the supernatant of each experiment was collected and stored at 4 °C to determine cytotoxicity. Triton X (0.2% in unsupplemented RPMI) was used for cell lysis as a positive control. The supernatant of untreated cells was used as negative control. The LDH assay was performed with the Cytotoxicity Detection Kit (Roche Applied Science, Mannheim, Germany) according to the supplier's manual.

### Trypan blue exclusion assay

The assay was carried out according to the manufacturer’s manual (Sigma Aldrich, Steinheim, Germany). Trypan was added to untreated cells and cells that had been exposed to NPs for 24 h. At a dilution of 1:10 in trypan blue, cells were stained and counted in a Neubauer chamber (Blau Brand, Ref. 717805, Wertheim, Germany). The positive control was performed by adding 0.2% Triton X onto the cells for 5 min, prior to adding trypan blue.

### Statistical analysis

The numerical data produced by the ImageJ macro scripts was exported into comma-separated values (CSV) files and imported into Microsoft Excel, where initial descriptive statistical values were calculated (mean, standard deviation). Finally, statistical analysis and plotting was performed in R [50]. Statistical significance was tested with Student’s t-test assuming an alpha value of 5%. The R script, with the hardcoded values, is provided in Supporting Information File 1.

## Supporting Information

File 1 Supporting Information contains in-depth descriptions of the experimental routines used in this manuscript.
