# Supplementary material for: Involvement of two uptake mechanisms of gold and iron oxide nanoparticles in a co-exposure scenario using mouse macrophages
Source: Beilstein J Nanotechnol. 2017 Nov 14;8:2396–409. doi: 10.3762/bjnano.8.239 (PMC5704759; doi:10.3762/bjnano.8.239)
Supplement: File 1 — Supporting Information contains in-depth descriptions of the experimental routines used in this manuscript. [file Beilstein_J_Nanotechnol-08-2396-s001.pdf]

Supporting Information

for

Involvement of two uptake mechanisms of gold and iron oxide nanoparticles in a co-exposure scenario using mouse macrophages

Dimitri Vanhecke[1], Dagmar A. Kuhn[1], Dorleta Jimenez de Aberasturi[2], Sandor Balog[1], Ana Milosevic[1], Dominic Urban[1], Diana Peckys[3], Niels de Jonge[3,4], Wolfgang J. Parak[2,5], Alke Petri-Fink[1]and Barbara Rothen-Rutishauser[*,§,1]

Address: [1]Adolphe Merkle Institute, Université de Fribourg, Chemin des Verdiers 4, CH 1700, Fribourg, Switzerland; [2]CIC Biomagune, Miramon Ibilbidea 182, 20014 Donostia, Gipuzkoa, San Sebastian, Spain; [3]Department of Biophysics, CIPMM Geb. 48, Saarland University, 66421 Homburg/Saar, Germany, [4]INM - Leibniz Institute for New Materials, Campus D2 2, 66123 Saarbrücken, Germany and [5]Fachbereich Physik, Philipps Universität Marburg, Renthof 7, 35037 Marburg, Germany

Email: Barbara Rothen-Rutishauser* - barbara.rothen@unifr.ch

* Corresponding author

§ Phone: +41 26 300 9502

Additional experimental data

Gold nanoparticles (AuNPs)

Synthesis of AuNPs

The gold nanoparticles (AuNPs) dissolved in organic media used in this work were synthesized according to the Brust two-phase method [1]. The description of the AuNP synthesis in this Supporting Information is adopted from previous publications [2,3]. Briefly, the aqueous gold precursor was freshly prepared by dissolving 0.300 g (0.9 mmol) hydrogen tetrachloroaurate(III) (99.9%, Alfa Aesar #12325) in 25 mL of Milli-Q water. In addition, a solution of 2.170 g (3.9 mmol) of tetraoctylammonium bromide (TOAB, Sigma-Aldrich #294136) in 80 mL of toluene (Fluka #89682) was directly prepared.

Both solutions were mixed and shaken vigorously for about 5 min in a separatory funnel. The $AuCl_4^-$ ions were gradually transferred into the organic phase (toluene) through the formation of tetraoctylammonium-gold tetrachloroaurate ion pairs.

$$AuCl_4^- + N(C_8H_{17})_4^+ \rightarrow N(C_8H_{17})_4^+AuCl_4^-$$

Once both phases were separated, the aqueous solution was then carefully discarded, and the toluene solution was transferred into a 250 mL round-bottomed flask. A freshly prepared solution of 0.334 g (8.8 mmol) of sodium borohydride ($NaBH_4$, Sigma #452882) dissolved in 25 mL of Milli-Q water was added dropwise to the vigorously stirred solution of gold precursor in toluene. The gold was then reduced by the sodium borohydride, and a change of colour from orange to violet was observed.

$$n\mathrm{AuCl}_4^- + 3ne^- \rightarrow 4n\mathrm{Cl}^- + \mathrm{Au}_n$$

Further growth was mediated due to the residue of sodium borohydride, which reduced the remaining gold ions. After one hour stirring, the solution was transferred again to a separatory funnel. A 25 mL solution of 0.01 M HCl was then added in order to remove the excess sodium borohydride. The mixture was vigorously shaken and the aqueous phase was discarded. Then, 25 mL of 0.01 M NaOH was added in order to remove the excess acid, and finally, four 25 mL

aliquots of Milli-Q water were added to remove the ion excess. Afterwards, the aqueous phase was discarded and the organics were transferred to a 250 mL round-bottomed flask. The solution was stirred overnight.

In order to obtain particles capped with 1-dodecanethiol (DDT), a ligand exchange step was carried out. Thus, 10 mL (8.450 g, 41.7 mmol) of DDT (Sigma #471364) were added, and the mixture was then heated at 65 °C for 3 hours. Due to the well-known high binding affinity of thiol to gold, DDT displaced the Br^- ions from the NP surface, obtaining DDT-coated AuNPs.

The solution was cooled down and larger agglomerates were removed by centrifugation at 670 g, collecting the supernatant. The obtained AuNPs were precipitated with methanol via selective size screening by a precipitation-re-dispersion mechanism that also removes the excess of DDT that could be remaining after the ligand exchange mechanism. After discarding the supernatant several times, the precipitate containing the hydrophobic AuNPs was dissolved in chloroform to store it for further use.

Characterization of AuNPs

In order to confirm the success of the synthesis, the AuNPs were characterized by transmission electron microscopy (TEM) and UV–vis absorption spectrometry. TEM images were collected with a JEOL JEM-1400PLUS transmission electron microscope operating at 120 kV, using carbon-coated 400 square mesh copper grids (CICbiomaGUNE, Spain). UV–vis absorption spectra were recorded using an Agilent 8453 UV-vis diode-array spectrophotometer. The average diameter of the inorganic core of the AuNPs, d_c, was determined by transmission electron microscopy (TEM) to be 5 ± 2 nm (Figure S1).

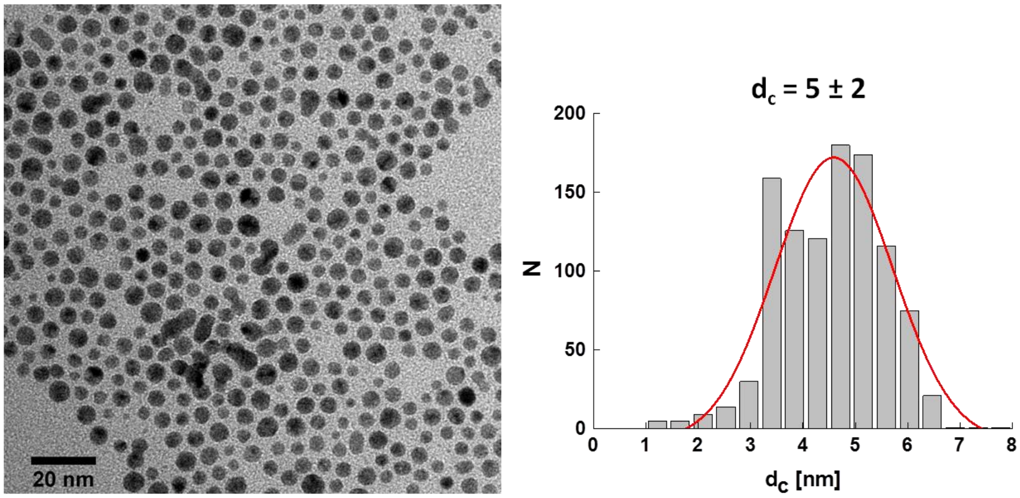

Figure S1: TEM image and the corresponding size distribution $N(d_c)$ of the core AuNPs. The average core diameter corresponds to $d_c = 4.7 \pm 2$ nm. The scale bar corresponds to 20 nm.

The AuNP concentration (c_{NP}) was determined from the UV–vis absorption using the Beer–Lambert law (Equation S1):

$$c_{NP} = A/(\varepsilon \cdot x \cdot l) \tag{S1}$$

In Equation S1, A is the absorption maximum at the surface plasmon resonance peak of the AuNPs, ε corresponds to the molar extinction coefficient at the wavelength of the absorption peak [$M^{-1}cm^{-1}$], l is the path length of the used cuvette [cm], and c_{NP} corresponds to the NP concentration of the sample [M].

In Figure S2 an UV–vis absorption spectrum is shown. The molar extinction coefficient (ε) used here was taken from a previous publication with very similar average diameter of the inorganic core d_c of the AuNPs [3] as $\varepsilon = 8.7 \times 10^6$ $M^{-1}cm^{-1}$, which was calculated following the method proposed by Liu et al. [4] using the parameters k = 3.3 and a = 10.80505. The obtained concentration of AuNPs was c_{NP} = 1 µM.

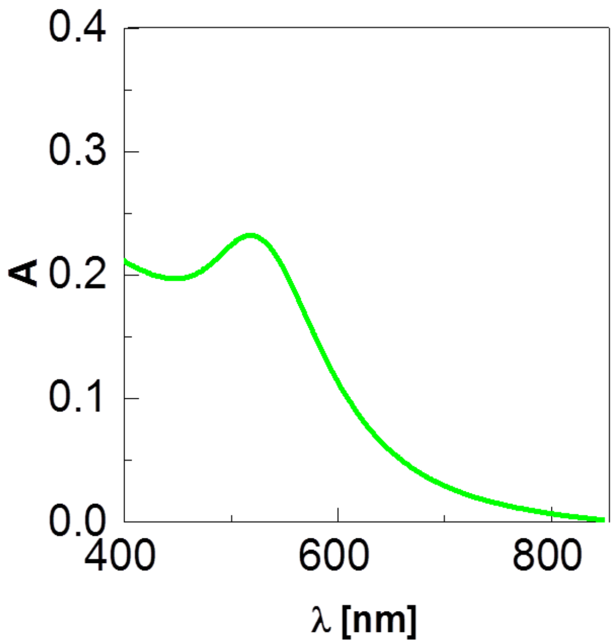

Figure S2: UV–vis absorption spectrum $A(\lambda)$ of AuNPs dissolved in chloroform.

Iron oxide nanoparticles (FeO_xNPs)

Synthesis of FeO_xNPs

Iron oxide nanoparticles were synthesized according to the method described by Hyeon and co-workers [5]. Briefly, 10 mL of dioctylether (Sigma-Aldrich #249599) mixed with 1.28 g of oleic acid (Sigma-Aldrich #O1008) was degassed at 60 °C for 20 min in a 50 mL round-bottomed flask. Afterwards, the solution was heated to 100 °C and 0.24 mL of $Fe(CO)_5$ (Sigma-Aldrich #481718) was added. The solution was heated to 295–300 °C and held at this temperature for 1.5 h. A condenser was used to prevent the evaporation of the solvent. After one hour, the mixture was cooled to room temperature. Then, 0.34 g of dehydrated trimethylamine oxide (Sigma-Aldrich #317594) was added, and the temperature was increased to 130 °C. The solution was kept at this temperature for two hours. After two hours, the temperature of the solution was increased with a ramp of 15 °C/min, until reaching the final temperature of 295 °C. The solution was then kept at this temperature for 1.5 h. The reaction was then stopped by removing the heating mantle. After cooling to room temperature, the NPs were purified via selective size screening by a precipitation–redispersion mechanism. Thereby, 2–5 mL of toluene was added, followed by 25–30 mL of methanol and the mixture was centrifuged at 440*g*. After several washes the final precipitate was re-dispersed in chloroform. Note, that no analytics were performed to verify the iron/oxygen stoichiometry and thus the NPs are referred to as FeO_xNPs.

Characterization of FeO_xNPs

Directly after synthesis and before transfer to aqueous solution, the FeO_xNPs were characterized by TEM and UV–vis absorption spectroscopy. The average diameter of the inorganic core of the FeO_xNPs, d_c, was determined by TEM to be 13.6 ± 4 nm (Figure S3).

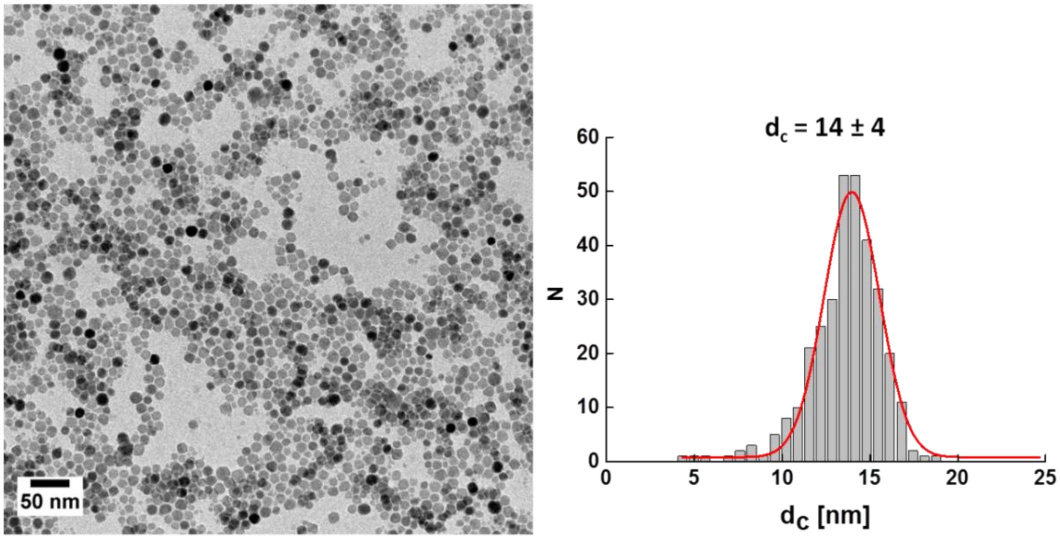

Figure S3:. TEM image and the corresponding size distribution $N(d_c)$ of the cores of the FeO_xNPs. The average core diameter corresponds to $d_c = 14 \pm 4$ nm. The scale bar corresponds to 50 nm.

In Figure S4, a UV–vis absorption spectrum is shown.

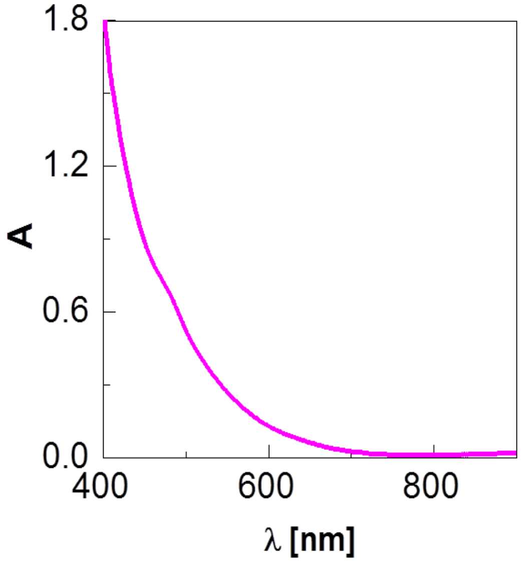

Figure S4: UV–vis absorption spectrum $A(\lambda)$ of FeO_xNPs dissolved in chloroform.

The concentration of the FeO_xNPs can be calculated by different methods. For example, concentration determination of FeO_x solution (in chloroform) can be obtained by weighing the sample. Related calculations are shown below:

- $c_{NP} = (N_{NP}/N_A)/V_{NP\ solution}$ with N_{NP} = number of NPs dispersed in volume $V_{NP\ solution}$

- N_A=6.022·10^{23} mol^{-1} = Avogadro's constant
- $N_{NP} = (m_{NP\ powder}/M_{NP})\cdot N_A$
- $m_{NP\ powder} = (m_{flask+NP\ powder} - m_{flask})$ = weight of NPs in dried NP solution of volume $V_{NP\ solution}$
- $M_{NP} = \rho_{NP}\cdot V_{NP}\cdot N_A$ = molar weight of FeO_xNPs (neglecting the organic surface capping)
- ρ_{NP} = 5.242 g/cm^3 = 5.242·10^{-21} g/nm^3 = density of FeO_x
- $V_{NP} = (4/3)\cdot\pi\cdot(d_c/2)^3$ = volume of the inorganic core of one FeO_xNP
- d_c = 13.6 ± 4 nm diameter of the inorganic core of one FeO_xNP as determined with TEM

This finally results in:

$c_{NP} = (N_{NP}/N_A)/V_{NP\ solution} = (((m_{NP\ powder}/M_{NP})N_A)/N_A)/V_{NP\ solution} = (m_{NP\ powder}/M_{NP})/V_{NP\ solution}$

$= ((m_{flask+NP\ powder} - \mathrm{m}_{flask})/(\rho_{NP}\cdot V_{NP}\cdot N_A))/V_{NP\ solution}$

$= (m_{flask+NP\ powder} - m_{flask})/V_{NP\ solution}/(\rho_{NP}\cdot(4/3)\cdot\pi\cdot(d_c/2)^3)/N_A$

$= (13.2667\ \mathrm{g} - 13.2587\ \mathrm{g})/5\ \mathrm{mL}/(5.242\cdot10^{-21}\ \mathrm{g/nm^3}\cdot(4/3)\cdot\pi\cdot(14\ \mathrm{nm}/2)^3)/6.022\cdot10^{23}\ \mathrm{mol^{-1}}$

$= 0.35\ \mu\mathrm{M}$

This concentration value corresponds to that obtained by measuring the absorbance of a 200 µL sample diluted to 2 mL of chloroform (A = 0.88; 1/10 dilution; see Figure S4) using a calculated molar extinction coefficient of ε = 1.3 × 10^7 M^{-1}cm^{-1} at 450 nm.

$c_{NP} = A\cdot10/\varepsilon/l = 0.88\cdot10/(1.3\times10^7\ \mathrm{M^{-1}cm^{-1}})/1\ \mathrm{cm} = 0.68\ \mu\mathrm{M}$

The values agree reasonably. In the following sections the concentration of FeO_xNPs has been calculated by measuring absorbance, as this method is easier to perform when the NPs are polymer-coated and dispersed in water.

Amphiphilic polymer for polymer coating synthesis

NPs that are soluble in organic media can be transferred to aqueous solution using an amphiphilic polymer. Thus, hydrophobic DDT-capped AuNPs and oleic acid-capped FeO_xNPs, both dissolved in chloroform, were transferred into aqueous solution using polymer-coating with an amphiphilic polymer based on maleic anhydride rings as backbone. Moreover, the selected polymer provides the possibility to attach other molecules containing free amino groups to the maleic anhydride rings. Thus, in this case, two different fluorophores, DY505 and DY615, which emit at different wavelengths, were used to label both types of NPs to easily differentiate them in vitro.

Polymer synthesis

The amphiphilic polymer used in this work was poly(isobutylene-*alt*-maleic anhydride)-*graft*-dodecylamine (PMA). The polymer was synthesized following a previously reported procedure [6,7]. The description of this procedure in this Supporting Information is adapted from these previous publications [2,3].

In summary, 2.70 g (15 mmol) of dodecylamine (98%, Sigma, # D22,220-8) was dissolved in 100 mL of anhydrous tetrahydrofuran (THF, ≥99.9%, Aldrich, #186562). This solution was poured into a 250 mL round-bottomed flask containing 3.084 g (20 mmol expressed as monomer) of poly(isobutylene-*alt*-maleic anhydride), average Mw ≈ 6,000 g/mol (Sigma, #531278). After mixing, the solution was sonicated for a few seconds (ca. 20 s) and heated to 60 °C for three hours. Afterwards, the solution was concentrated to 30–40 mL and the mixture was heated under reflux overnight. Finally, the solvent was completely evaporated and the polymer powder was re-dissolved in 40 mL anhydrous chloroform to obtain a final (monomer) concentration of c_P = 0.5 M. With this ratio of dodecylamine and poly(isobutylene-*alt*-maleic anhydride), 75% of the anhydride rings reacted, leaving 25% of these groups for further modification, in this case dye incorporation.

Figure S5 shows the structure of the poly(isobutylene-*alt*-maleic anhydride) hydrophilic backbone modified with dodecylamine (PMA).

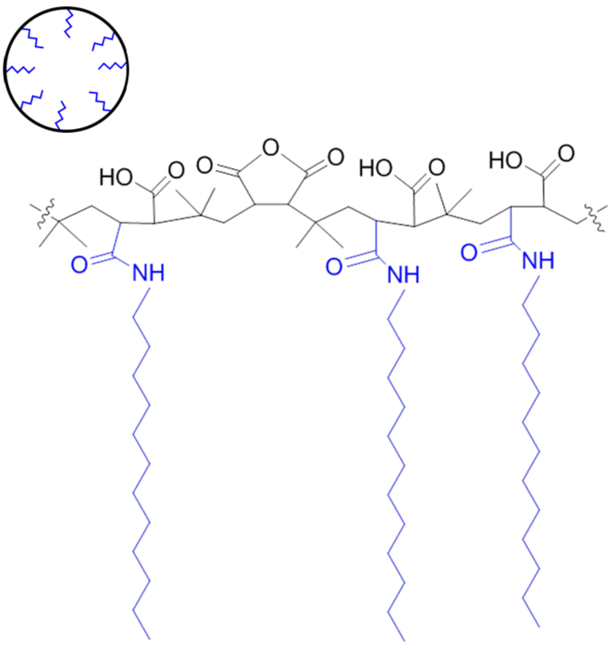

Figure S5. Structure of the amphiphilic polymer (PMA). The polyisobutylene-*alt*-maleic anhydride hydrophilic backbone (black) is modified with dodecylamine hydrophobic side chains (blue). Reproduced with permission from [2], copyright 2014 AIP publishing.

Polymer functionalization with organic fluorophores DY505 and DY615

Two different dyes were selected to label the NPs. DY505 (amino derivative DY505, absorption maximum at 505 nm, emission maximum at 530 nm in ethanol, dyomics, #505-02), and DY615 (amino derivative DY615, absorption maximum at 621 nm, emission maximum at 641 nm in ethanol, dyomics, #615-02). Both were linked via amide bonds to their maleic anhydride rings [6,8]. According to previous reports [9,10], 2% of the total anhydride rings of the amphiphilic polymer were modified by the reaction of the maleic anhydride rings of the amphiphilic polymer with the amino group of the corresponding dye. 1 mg of the dissolved dye was mixed with 200 μL of a 0.5 M sample (in terms of monomer concentration) of the prepared amphiphilic polymer solution. The reaction mixture was refluxed overnight at room temperature. The mixture was evaporated and the dye-modified polymer was re-dissolved in anhydrous chloroform to obtain a final (monomer) concentration c_P = 0.05 M (Figure S6).

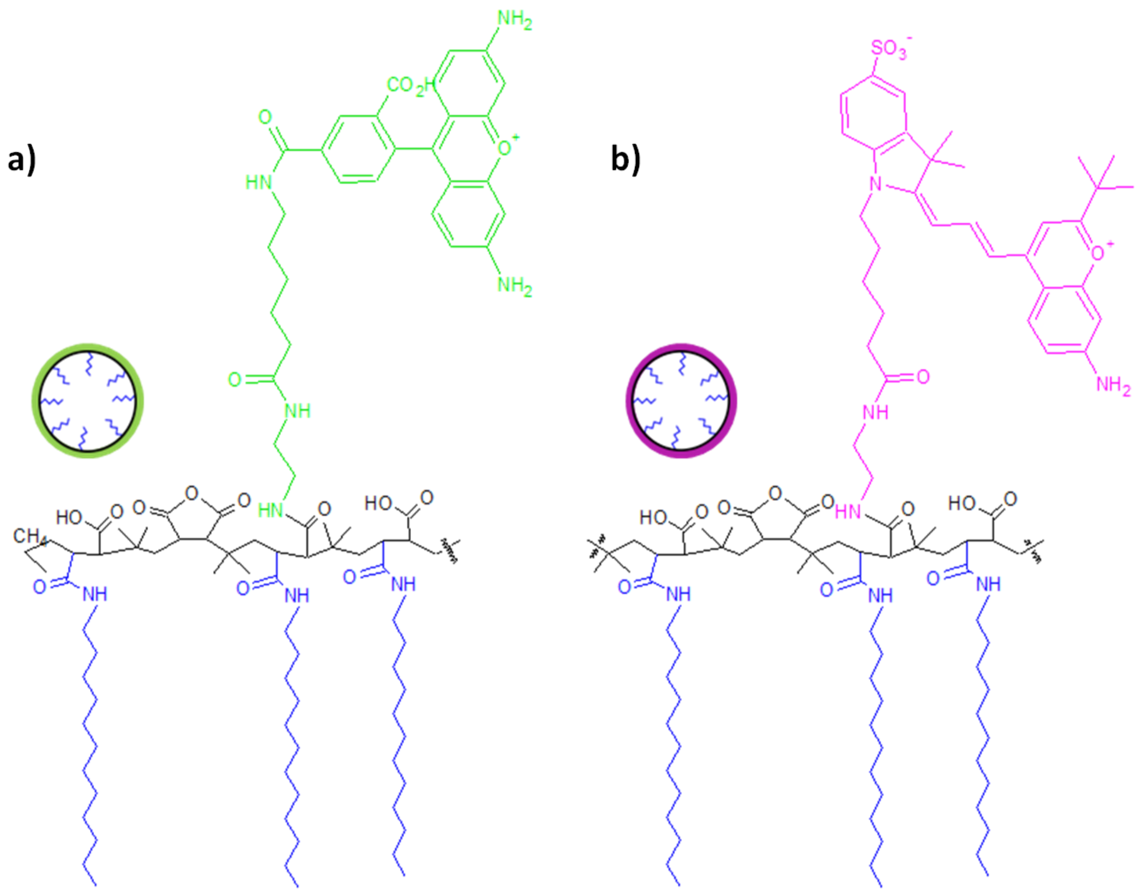

Figure S6: Scheme of the linkage of a) amine-DY505 (green) b) amine-DY615 (pink) via amide bonds to the anhydride rings of the hydrophilic backbone of the amphiphilic polymer (black).

Polymer coating

As previously reported, the amount of polymer required for coating of the NPs was determined by Equation S2:

$$V_p = \frac{\pi \cdot c_{NP} \cdot V_{NP\ solution} \cdot d_{eff}^2 \cdot R_{p/area}}{c_p}. \quad \text{(S2)}$$

The variables c_{NP} and $V_{NP\ solution}$ correspond to the NP concentration and volume of the NP solution. c_p and V_p represent the monomer concentration of the amphiphilic polymer dissolved in chloroform and the volume of the polymer solution. d_{eff} is the effective diameter of the NPs: $d_{eff} = d_c + 2 \cdot l_{surfactant}$ = the sum of the diameter of the inorganic core (d_c) plus the assumed thickness of the surfactant shell. We assumed that $l_{surfactant} \approx 1$ nm. $R_{p/area}$ is the number of polymer monomers which need to be added per surface area of NPs in monomer units/nm^2. The effective surface area of one single NP is $A_{eff} = 4\pi \cdot (d_{eff}/2)^2$.

Polymer coating of Au nanoparticles with the DY505-functionalized polymer

AuNPs (d_c = 5 nm ⇒ d_{eff} = 5 nm + 2·1 nm = 7 nm; $V_{NP\ solution}$ = 2000 µL, c_{NP} = 1 µM in chloroform) were mixed with PMA–DY505-2% in chloroform (V_P = 308 µL, c_P = 0.05 M, $R_{p/area}$ = 50 nm^2) in a 25 mL round-bottomed flask. The mixture was stirred and the solvent was slowly evaporated. The resulting solid film containing the NPs was dissolved in 28 mM sodium borate buffer at pH 12 (SBB 12). The basic pH value of the buffer induces the opening of the remaining anhydride rings, leading to negatively-charged carboxylate groups at that pH value, which increases the surface charge and renders the NPs water-soluble.

Polymer coating of Fe nanoparticles with DY615-functionalized polymer

Following the same procedure as for AuNPs, FeO_xNPs (d_c = 14 nm ⇒ d_{eff} = 14 nm + 2·1 nm = 16 nm; $V_{NP\ solution}$ = 2000 µL, c_{NP} = 0.74 µM in chloroform) were mixed with PMA–DY615-2% in chloroform (V_P = 1905 µL, c_P = 0.05 M, $R_{p/area}$ = 80 nm^{-2}) in a 25 mL round-bottomed flask. The mixture was stirred and the solvent was slowly evaporated. The resulting solid film containing the NPs was dissolved in 28 mM sodium borate buffer at pH 12 (SBB 12).

Purification and characterization of polymer-coated nanoparticles

After polymer-coating, both NP samples were purified and characterized using different techniques.

Gel electrophoresis

Gel electrophoresis was used to purify the sample and eliminate empty polymer micelles. For this purpose, 2% agarose gels were prepared by dissolving the agarose with tris-borate-EDTA buffer (TBE; 0.5×). The concentrated NP samples were mixed with glycerol to increase the density and were directly placed in the pre-prepared gel. Applying an electric field of 15 $V \cdot cm^{-1}$ the negatively-charged NPs migrated towards the positive pole. After an hour of running time the polymer-coated NPs formed a band that was separated from the band of the empty micelles or free dyes, as can be observed in Figure S7. The NPs were cut out of the gel and placed into a dialysis membrane (50 kDa weight cut-off (MWCO)) filled with TBE buffer. After applying an electric field again for 20 min, the NPs emerged from the gel and stayed trapped inside the dialysis membrane. In order to be sure that all empty micelles were removed, this procedure was applied twice.

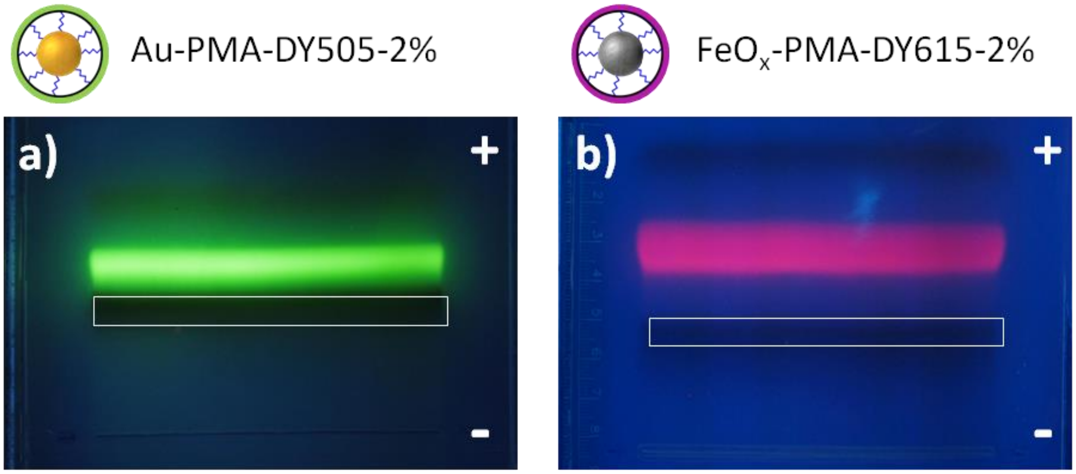

Figure S7: Image of the gels under UV light. a) Polymer-coated AuNPs (Au-PMA-DY505-2%), which can be distinguished from the empty polymer micelles (green). b) Polymer-coated FeO_xNPs (FeO_x-PMA-DY615-2%), which can be distinguished from the empty polymer micelles (pink). The images of the gels were taken after 60 min running time within an electric field of

15 Vcm^{-1}. "+" and "–" indicate the polarity of the electrodes with which the electric field was applied.

After extracting the NPs, they were washed and concentrated using a centrifuge filter (*MWCO* = 100 kDa, Sartorius Stedim, 1320*g* centrifugation speed).

NP characterization

Ultraviolet–visible absorption spectroscopy

The concentration of the NPs was again determined by UV–vis absorption measurements (Figure S8).

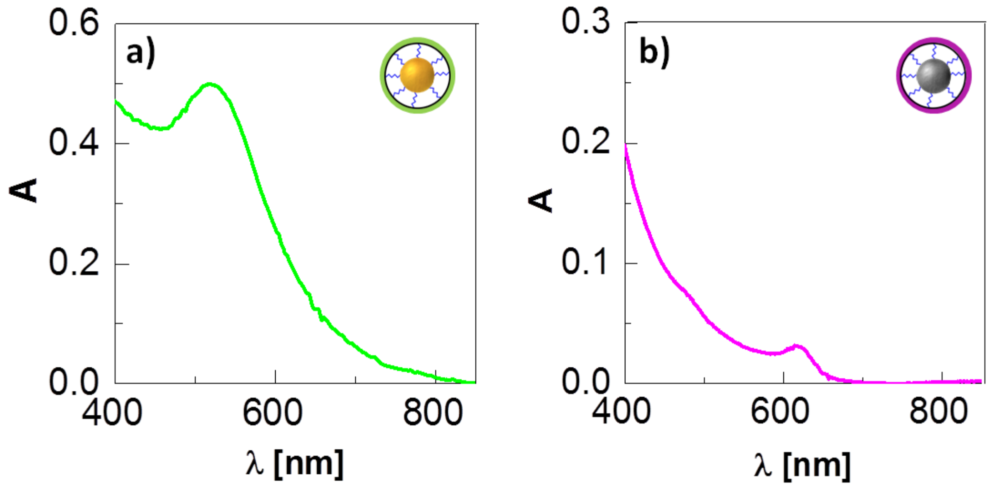

Figure S8. UV–vis absorption spectra of a) Au-PMA-DY505-2% and b) FeO_x-PMA-DY615-2% dissolved in water. The absorption *A* is plotted as a function of the wavelength λ. In the case of the FeO_xNPs the peak in the absorption spectrum is due to the absorption of the DY615 fluorophore, which is incorporated into the polymer shell.

Fluorescence spectroscopy

Fluorescence measurements were recorded using a Fluorolog-3 apparatus (model FL3-22, Horiba). The emission spectra of both samples are shown in Figure S8. Au-PMA-DY505-2% NPs were excited at 505 nm and FeO_x-PMA-DY615-2% NPs were excited at 600 nm (Figure S9).

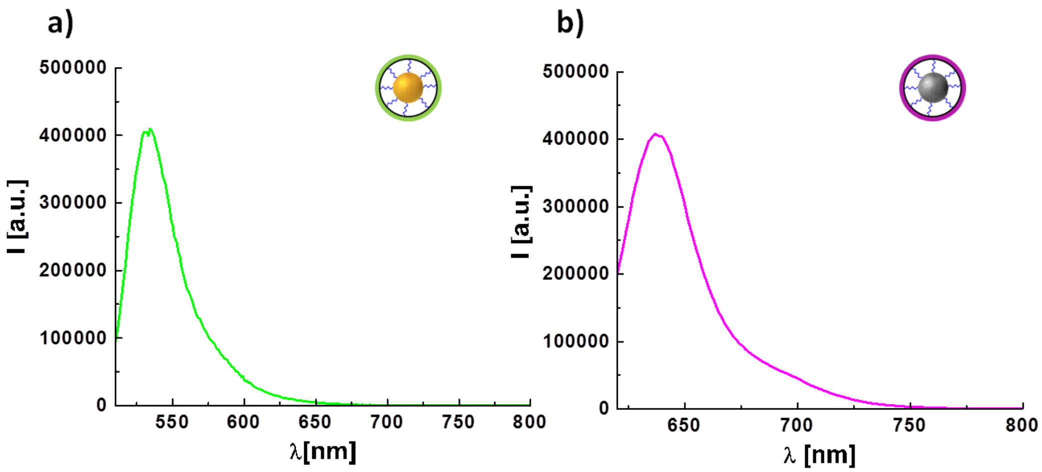

Figure S9: Fluorescence emission spectra of a) Au-PMA-DY505-2% and b) FeO_x-PMA-DY615-2% NPs recorded in water. The fluorescence intensity *I* is plotted as a function of the wavelength λ. In both cases fluorescence is due to the fluorophores that are incorporated into the polymer shell around the NP cores.

TEM images of polymer-coated NPs (Figure S10)

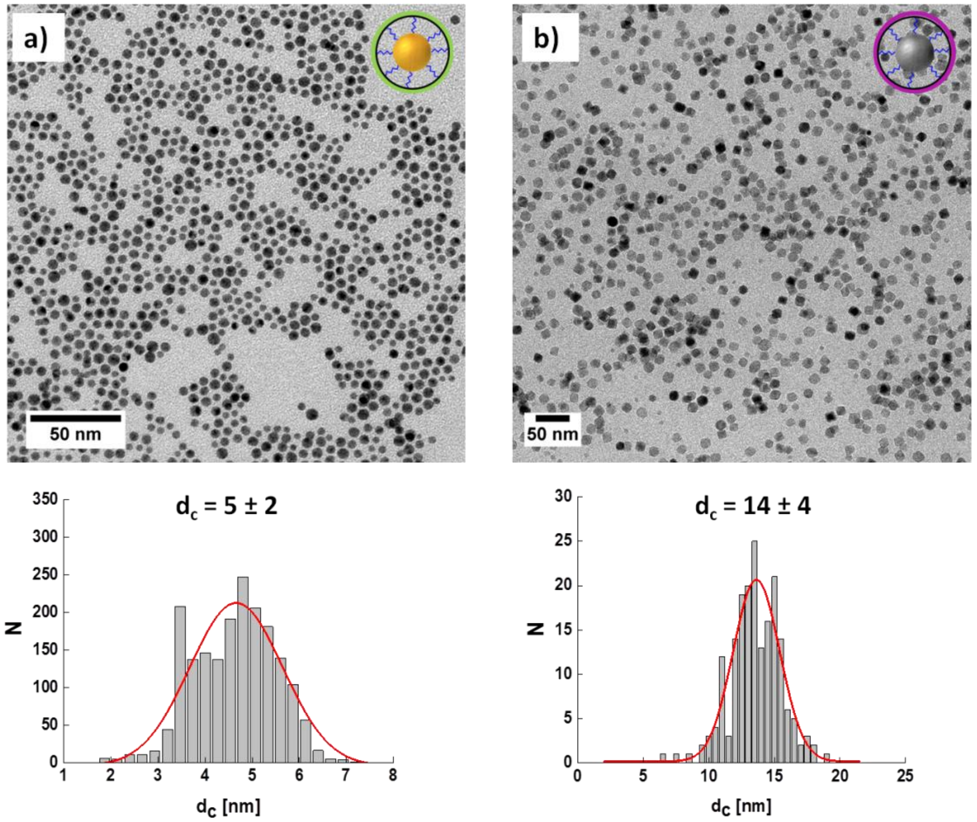

Figure S10: TEM image and the corresponding size distribution $N(d_c)$ of the core of a) Au-PMA-DY505-2% and b) FeO_x-PMA-DY615-2% NPs. The scale bar corresponds to 50 nm.

Determination of hydrodynamic diameter and zeta potential

The hydrodynamic diameter, d_h, of the NPs was determined via dynamic light scattering (DLS) and the zeta potential (ζ) of the different NPs were calculated via laser doppler velocimetry (LDV) using a ZetasizerNano ZS apparatus (Malvern Instruments). All of the samples were equilibrated to 25 °C for 3 min before measuring. The obtained values were calculated as an average of three measurements with their corresponding standard deviations (Figure S11, Figure S12).

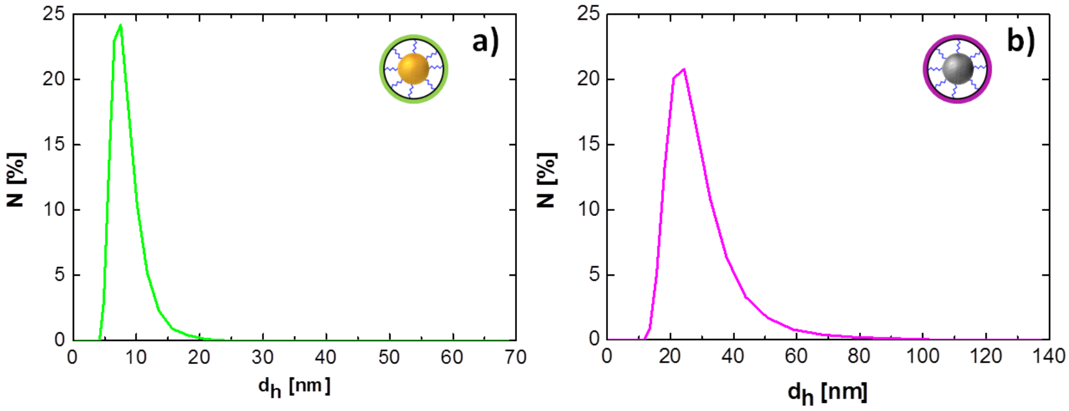

Figure S11: Number distribution N(%) of the hydrodynamic diameter d_h of a) Au-PMA-DY505-2% and b) FeO_x-PMA-DY615-2% NPs measured in water. The d_h of Au-PMA-DY505-2% was found to be 11 ± 3 nm and that of FeO_x-PMA-DY615-2% was found to be 28 ± 9 nm.

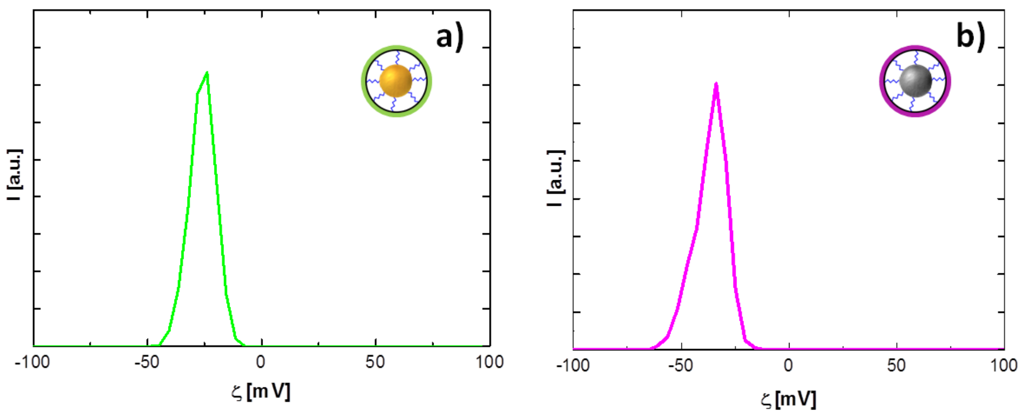

Figure S12: Distribution I of the zeta potential ζ of a) Au-PMA-DY505-2% and b) FeO_x-PMA-DY615-2% NPs. The mean values of the zeta potentials as determined in water correspond to −26 ± 2 mV for Au-PMA-DY505-2% and −37 ± 1 mV for FeO_x-PMA-DY615-2%.

Determination of the number of dye molecules bound per NP

The number of dye molecules bound to each NP was estimated for the FeO_xNPs (d_c = 14 nm) and for the AuNPs (d_c = 5 nm) (Figure S13).

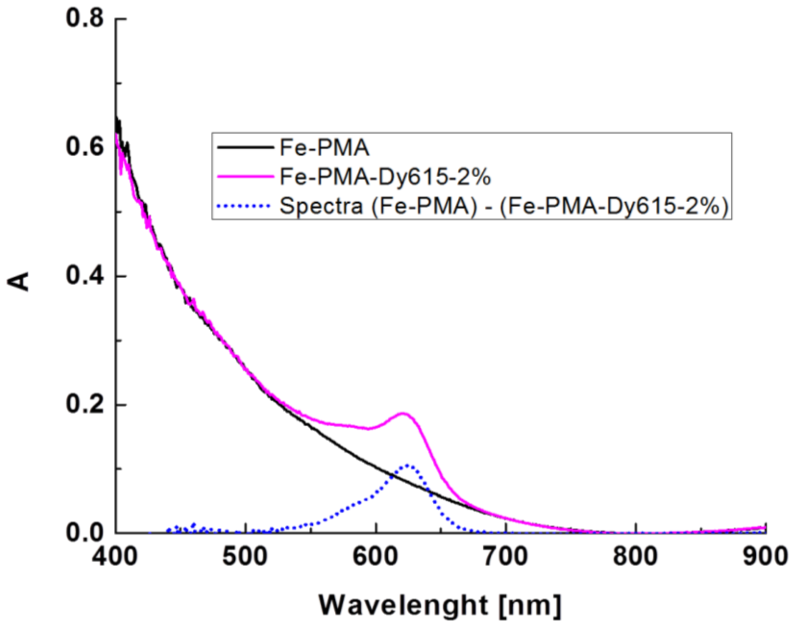

Figure S13: UV–vis spectra recorded for FeO_x-PMA-DY615-2% (pink), FeO_xNPs coated with plain polymer without integrated fluorophore (FeO_x-PMA) (black) and subtraction of UV–vis spectrum of FeO_x-PMA from the spectrum of FeO_x-PMA-DY615-2% (blue).

To determine the number of DY615 fluorophores bound per FeO_xNP, the dye concentration in a solution of FeO_x-PMA-DY615-2% NPs was calculated using the DY615 dye absorption at its absorption maximum, and the iron oxide NP concentration was determined by the absorption of the sample at 450 nm. From both concentrations (dye concentration, NP concentration), the number of attached dye molecules per NPs was derived.

As it was observed that the FeO_xNPs themselves do not present a characteristic peak in their absorption spectrum. For evaluation the absorption spectra were first background-corrected by subtracting the absorption at 800 nm, which is considered as a constant offset. Then, both spectra were normalized to the absorption at 450 nm. In order to determine the contribution of the dye absorption in the absorption spectrum of the FeO_x-PMA-DY615-2% NPs, the absorption spectrum of the plain FeO_xNPs was subtracted from the spectra of FeO_xNPs modified with the polymer with integrated dye. From the absorption spectra the concentrations were determined using the Beer–Lambert law (Equation S1)

DY615 dye has an absorption maximum at around 615 nm and the extinction coefficient at this wavelength is ε(615 nm) = 200,000 $M^{-1}cm^{-1}$ (provided by the supplier). The extinction coefficient of the FeO_xNPs was assumed to be ε(450 nm) = 1.3 × 10^7 $M^{-1}cm^{-1}$. In this way the concentration of dye, based on the absorption at 615 nm, and the FeO_xNP concentration, based on the absorption at 450 nm, were determined. By dividing the dye concentration by the FeO_xNP concentration, the dye/FeO_xNP ratio was finally calculated [52]. The pathlength of the cuvette for absorption measurements was l = 1 cm. For the NP and dye absorption the following values were measured:

A(FeO_xNP @ 450 nm) = 0.38
A(dye @ 615 nm) = 0.10

According to the Beer–Lambert law this leads to the following concentrations:

$c_{NP} = A(FeO_xNP\ @\ 450\ nm)/(l \cdot \varepsilon(450\ nm)) = 0.38/(1\ cm \cdot 1.3 \times 10^7\ M^{-1}cm^{-1}) = 2.9 \times 10^{-8}\ M$
$c_{dye} = A(dye\ @\ 615\ nm)/(l \cdot \varepsilon(615\ nm)) = 0.10/(1\ cm \cdot 2 \times 10^5\ M^{-1}cm^{-1}) = 5.1 \times 10^{-7}\ M$

Thus, the number of fluorophores bound per FeO_x-PMA-DY615-2% NP is $N_{dye/NP} = c_{dye}/c_{NP}$ = 5.1×10^{-7} M/2.9×10^{-8} M ≈ 17.4

In the case of the Au-PMA-DY505-2% NPs, the absorption band of the dye was not distinguishable from the plasmon peak of the AuNPs at 520 nm (cf. Figure S8a). Thus, it was not possible to calculate the concentration of the dye using the absorption method as described for the FeO_xNPs. Therefore, an estimation of the number of dyes bound to each AuNP was made by scaling the number of fluorophores per NP by the size of the NP.

For scaling the following parameters were used:

- core radius of the NPs: r_c (= $d_c/2$)
- thickness of the first ligand shell (surfactant of the hydrophobic nanoparticles): $l_{surfactant} \approx$ 1 nm
- effective radius $r_{eff} = r_c + l_{surfactant}$ (= $d_{eff}/2$)

- estimation of the thickness of the first ligand shell and the amphiphilic polymer cap, as described in [11]: $l_{pol} \approx 4$ nm
- radius of one NP including the polymer shell $r_p = r_c + l_{pol}$
- hydrodynamic radius r_h (= $d_h/2$)
- surface areas: $A_{eff} = 4\pi \cdot r_{eff}^2$, $A_p = 4\pi \cdot r_p^2$
- number of fluorophores per NP: $N_{dye/NP}$

The precise location of the fluorophores in the polymer shell is unknown, but they will be located within a shell between r_{eff} (where the polymer coating begins) and r_p (where the polymer coating ends). The number of fluorophores per NP is assumed to be proportional to the surface area: $N_{dye/NP} \propto A \Rightarrow N_{dye/NP1}/N_{dye/NP2} = A_1/A_2 = r_1^2/r_2^2 = d_1^2/d_2^2$. Here, r can refer to r_{eff} or r_p and thus

$N_{dye/NP,eff,2} = N_{dye/NP,eff,1} \cdot A_{eff,2}/A_{eff,1}$
$N_{dye/NP,p,2} = N_{dye/NP,p,1} \cdot A_{p,2}/A_{p,1}$

Above we have calculated that for d_c = 14 nm FeO_xNPs there are $N_{dye/NP}$ = 17.4 dyes/NP. Based on this, it was possible to estimate the number of fluorophores per AuNP with d_c = 5 nm in the following way:

The parameters for FeO_xNPs are**:** $d_{c,1}$ = 14 nm $\Rightarrow$ $d_{eff,1}$ = 14 nm + 2·1 nm = 16 nm; $d_{p,1}$ = 14 nm + 2·4 nm = 22 nm; $N_{dye/NP1}$ = 17.4.

For the AuNPs the following parameters are known: $d_{c,2}$ = 5 nm $\Rightarrow$ $d_{eff,2}$ = 5 nm + 2·1 nm = nm; $d_{p,2}$ = 5 nm + 2·4 nm = 13 nm.

Thus, if all fluorophores are assumed to be located at the inner surface, one obtains $N_{dye/NP,eff,2} = N_{dye/NP,1} \cdot r_{eff,2}^2/r_{eff,1}^2 = 17.4 \cdot (3.5\text{ nm})^2/(8\text{ nm})^2 \approx 3.3$.

If all fluorophores are assumed to be located at the outer surface one obtains
$N_{dye/NP,p,2} = N_{dye/NP,1} \cdot r_{p,2}^2/r_{p,1}^2 = 17.4 \cdot (6.5\text{ nm})^2/(11\text{ nm})^2 \approx 6.1$.

We then take the mean value of both situations, leading to $N_{dye/NP,2}$ = (3.3 + 6.1)/2 = 4.7 ± 1.4. We thus conclude that in the polymer shell of the AuNPs with d_c = 5 nm there are around $N_{dye/NP}$ = 5 ± 1 fluorophores per NP.

Profile plots on ESEM data

Below are two examples of linearity of NPs in the ESEM data (Figure S14). The figures are enlarged versions of the data in Figure 3. More such observations were made in this work. The NPs, clearly visible in white, stand out against the green cellular structure. Here, a profile plot along the box was produced. NPs appear as peaks, making it possible to correlate the number of peaks with the distance. The boxplot summarizes the distance between the peaks, along with their variation.

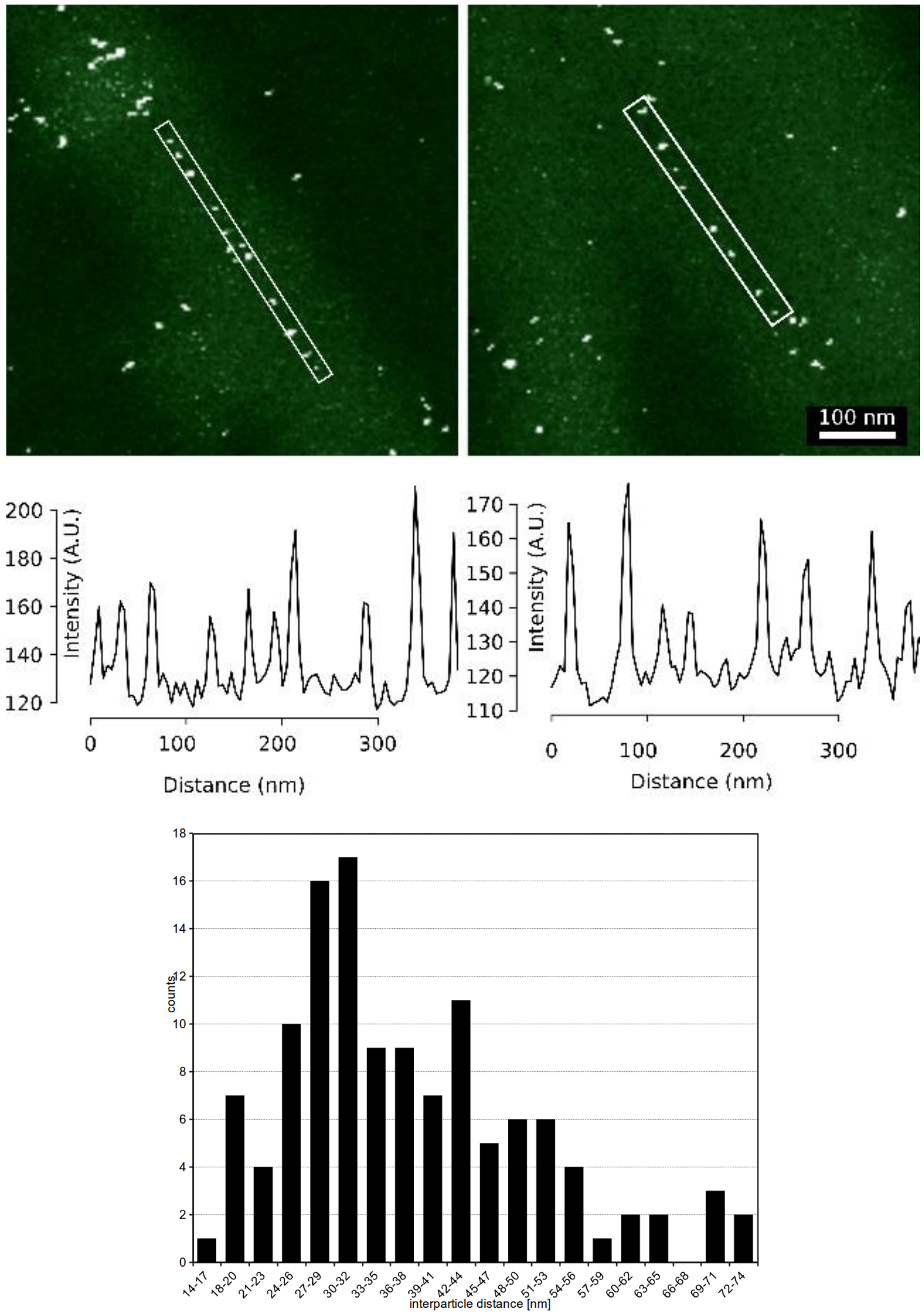

Figure S14: Images (top) and profile plots along seemingly linear composition of AuNPs. The peaks show the position of the AuNPs. The histogram of interparticle distance shows visually an average distance of 37 ± 13) nm between AuNPs.

LDH tests

Standard LDH tests for cytotoxicity were performed (Figure S15, Figure S16).

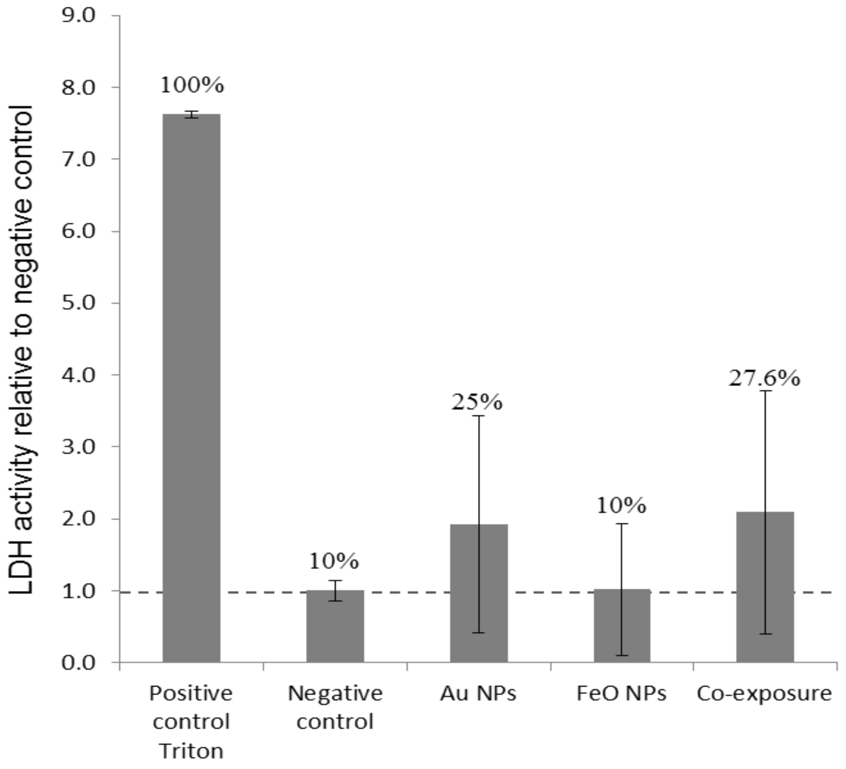

Figure S15: LDH activity was measured compared to the negative control for J774A.1 cells and percentages show the measured samples compared to the positive control (Triton X). J774A.1 cells that were exposed to Au and FeO_xNPs in single and co-exposure experiments for 24 h showed no increased value under either set of conditions. All measurements were performed in triplicate for all samples. Data are shown as mean value ± standard deviation (SD).

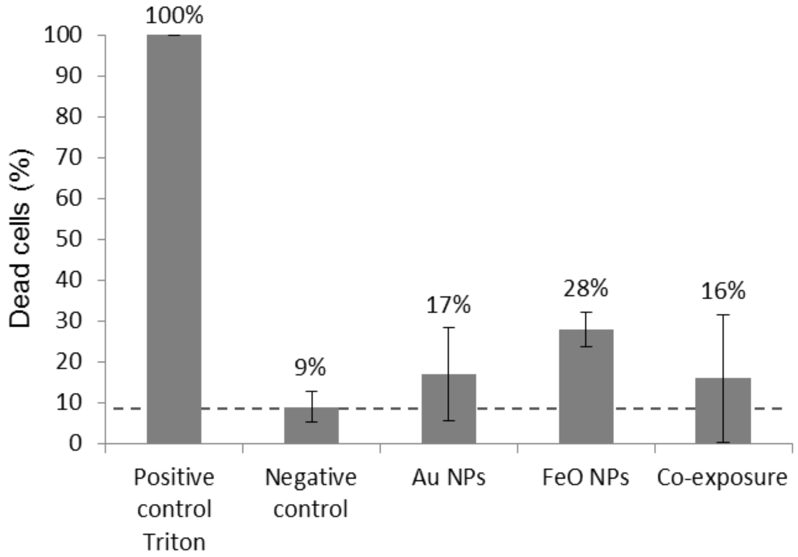

Figure S16: After 24 h of NP exposure, trypan blue was added at a dilution of 1:10 and the percentage of dead cells was counted. Compared to the negative control, the samples did not show an increased cell death rate. Data are given as mean ± SD.

Colocalization of NPs with lysosomes (Figure S17)

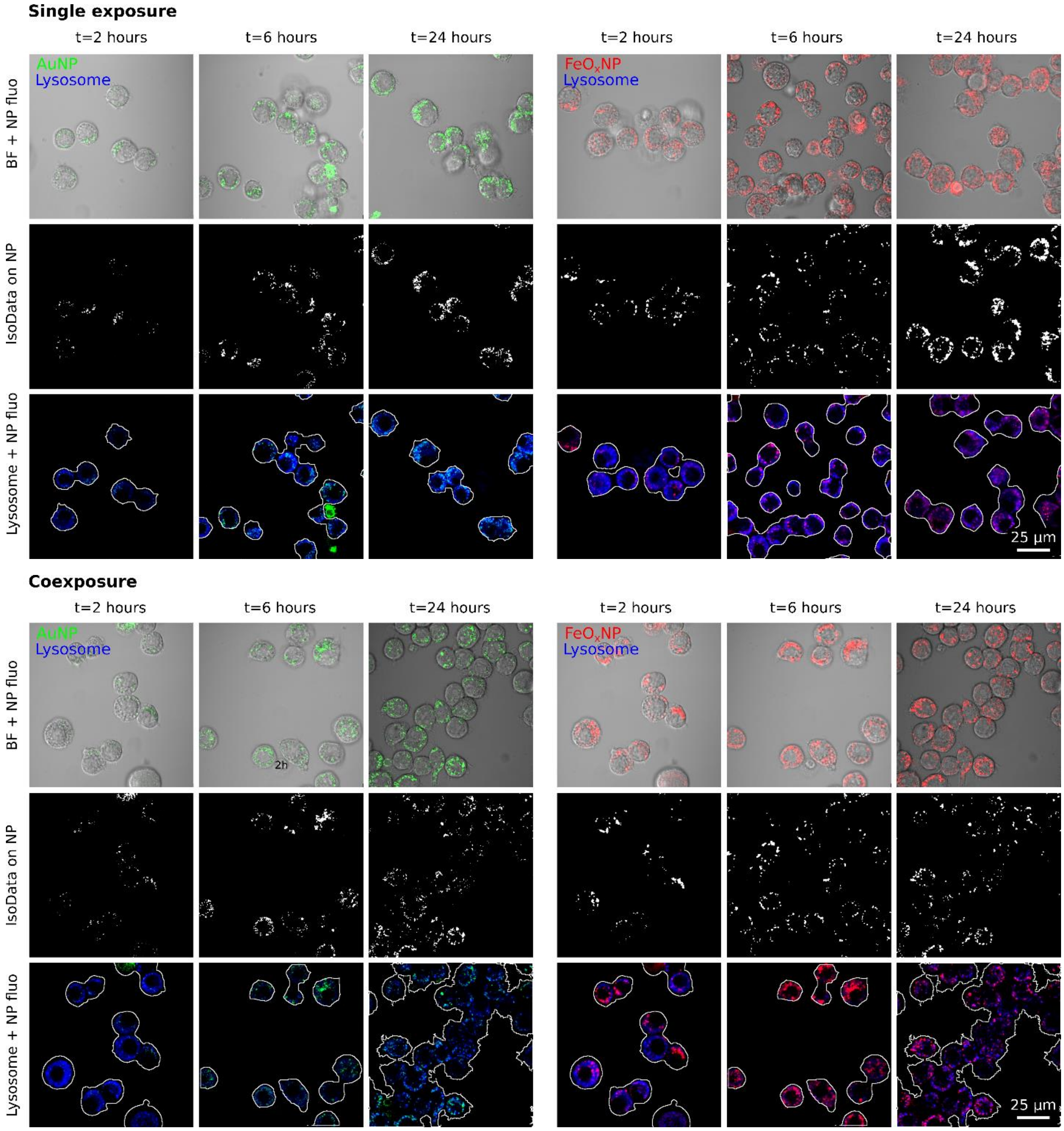

Figure S17: Top row: Brightfield channel overlaid with the fluorescence of the NP; i.e., green for AuNPs and red for FeO_xNPs. Middle row: IsoData binarization of the NP channel, yielding white pixels corresponding to pixels associated with the NP and black for all other pixels. Bottom row: Outline of the cells, based on the variance/mean filtering of the brightfield channel overlaid

with the lysosomal marker channel and the fluorescence of the NP (green for AuNP, red for FeO_xNP). Left column: 2 h exposure, middle column: 6 h exposure, right column: 24 h exposure. The panels are in four-fold: AuNP single exposure, FeO_xNP single exposure, AuNP co-exposure (not showing the FeO_xNP channel) and FeO_xNP co-exposure (showing only the FeO_xNP channel).

Quantification of monodansylcadaverine (MDC) effect

Quantification of markers in the vicinity of the cell is not straightforward for a number of reasons. (1) A possible comparison depends on the background and (2) "vicinity" is a vague description.

We circumvented the first issue by offering numbers relative to background values. The background fluorescence is measured at a sufficient large spot, at a distance from the cell. Next, we defined "vicinity" as relative to the cell profile area in every slice: the cell is masked using the actin stain, the mask is extended (scaled) to 120% and the original actin mask is subtracted. This yields a rim-like mask of 1–2 µm surrounding the cell (Figure S18). The scale factor (20%) is obviously subjective, a number based on visual inspection and size measurements of the aggregates in the single exposure. Too small scale factors suffer from imprecision of the actin mask (which could assess NPs during uptake as blocked) whereas too large scale factors record mostly background.

We opted for a relative scale factor and against a pure enlargement of the mask in absolute numbers since this would overestimate the vicinity around the poles of the cell (where the cell profiles are smaller).

We used the actin stain as a mask for the cell. Pixel values were multiplied by 2 (practically a 2-fold increase of brightness) in order to facilitate the conversion to a mask. The mask contains white pixels (positive) and black pixels (background). The expansion of the mask was carried out by creating a selection, and perform a centred scaling of that selection of 120% in *x*- and *y*-directions. This yields an enlarged mask of the actin stain. Finally, the initial actin mask is subtracted from the enlarged mask by a XOR method. The result is a mask that follows the rim of the cell.

This mask is calculated for each slice in the stack and overlaid with the corresponding AuNP and FeO_xNP signal. The average values of that calculation are presented as factor of the average background value some distance (more than 15 µm) away from the cell.

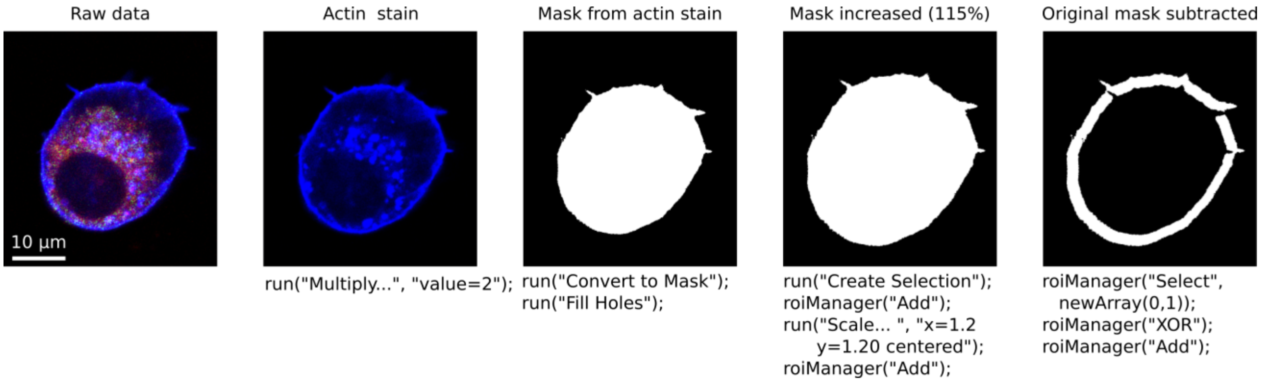

Figure S18: The procedure to quantify the inhibitory effect of MDC. From the actin stain, a mask is produced (white), the mask is expanded and from the expanded mask, the initial mask is subtracted.

Analytical data from fluorescence data

Note: remarks to better understand the script are written in red and preceded by a "//".

```
// Setup of data control. The data is arranged in a hierarchical folder system based on exposure length (2, 6 or 24 h) followed by subfolders describing the particle type (here: FeONP)

hours="24";
particle="FeONP";
dir="~/"+hours+"h/"+particle+"/";
list = getFileList(dir);
number_of_images=list.length;
setBatchMode(true); //This assures that the routines are performed without refreshing the video, making it much faster.

median_filter=1; //sets the strength of the median filter
//feed the order of the channels in the data to the script
source_channel="3"; // the brightfield channel
lookup_channelgreen="1"; // fluorescence channel 1
lookup_channelred="2"; // fluorescence channel 2

//start a loop through all images in the folder
for (image_number=1;image_number<=number_of_images;image_number++){
      filename = list[image_number-1];
```

```
	// open the data, retrieve its basic information, rename it "stack" and
	duplicate this stack to do the masking (this duplicate is called "calc")
	open(dir+filename);
	rename("stack");
	selectWindow("stack");
	getDimensions(width, height, channels, slices, frames);
	run("Duplicate...", "title=calc duplicate channels=3 slices=1-"+slices);

	// Perform the brightfield masking
	selectWindow("calc");
	run("Variance...", "radius=1 stack");
	run("Median...", "radius=2 stack");
	run("Variance...", "radius=1 stack");
	run("Median...", "radius=2 stack");
	setOption("BlackBackground", false);
	setAutoThreshold("Default");
	run("Convert to Mask", "method=Default background=Light calculate");
	run("Fill Holes", "stack");

	// Retrieve intensity values at each pixel inside the masked area: start
the loop through all images in the stack
for (e=1;e<=slices;e++){
	// Perform the masking of the fluorescence channels based on the mask
	retrieved from the brightfield channel
	if(roiManager("count")>0) {
		run("Select None");
		roiManager("Delete");
	}
	selectWindow("calc");
	setSlice(e);
	run("Create Selection");
	roiManager("Add");

	// find mean values in masks in both fluorescence channels, called
	"green" and "red" for ease here
	selectWindow("stack");
	setSlice(1+(3*(e-1))+0);
	roiManager("Select", 0);
	getStatistics(area, mean, min, max, std, histogram);
	avg_green=mean;
	setSlice(1+(3*(e-1))+1);
	roiManager("Select", 0);
	getStatistics(area, mean, min, max, std, histogram);
	avg_red=mean;
	run("Select None");
	selectWindow("calc");
	run("Select None");

	//reset the variables used for the pearsons colocalisation coefficient
	calculation (for nominator and denominator of the formula)
	g_denom=0;
	r_denom=0;
	nom=0;
	//Pearsons data collection
	for (W=0;W<width;W++){
		for (H=0;H<height;H++){
			selectWindow("calc");
			setSlice(e);
```

```
                    location = getPixel(W,H);

                          if (location==255) {
                                selectWindow("stack");
                                setSlice(1+(3*(e-1))+0);
                                p_green = getPixel(W,H);
                                setSlice(1+(3*(e-1))+1);
                                p_red = getPixel(W,H);
                                nom=nom+((p_green-avg_green)*(p_red-avg_red));
                                g_denom = g_denom + (pow(p_green-avg_green,2) );
                                r_denom = r_denom + (pow(p_red-avg_red,2) );
                          }
                  }
            }
}
// Calculation of the pearsons colocalisation coefficient
denom= sqrt(g_below * r_below);
pearsons=nom/denom;

// cleanup
selectWindow("stack");
close();
selectWindow("calc");
close();
```

In a similar way, one can retrieve the fluorescence value of each pixel after masking and IsoData classification, yielding the number of pixels and their values, marking NP uptake over time.

The authors are aware that use of bright-field to mask the fluorescence is potentially prone to bias, especially since bright-field data is not confocal. However, the main goal of this masking is not to delineate the cell at the highest resolution, the goal is to minimize background, which may bias the Pearson's coefficient. In this light, the bright-field masking technique provides robust results, as long as the field of view is not contaminated with dead cells.

R script for statistical analysis

```
#CONFIDENCE INTERVAL CALCULATIONM AND PLOTS

#==========================================

#ConfA= 95% confidence interval below the mean, ConfB = 95% confidence interval above the mean

#AuNP, single exposure

#AuNPx: Particle: AuNP or FeONP, x=(s) or (c)oexposure

AuNPsArea <- c(0.8967163069,1.2770011125,4.6871686257)

AuNPsAreaSD <- c(0.5017917241,0.965835508,2.4397364195)

AuNPsAreaConfA <- c(0.7707928413,1.0305531381,4.1774275111)

AuNPsAreaConfB <- c(1.0226397726,1.5234490869,5.1969097402)

AuNPsIntensity <- c(10443.3910152749,9981.0231362073,9368.1710055123)

AuNPsIntensitySD <- c(2967.4377527925,3102.0054057569,2289.5649811351)

AuNPsIntensityConfA <- c(9698.719415367,9189.4981378932,8889.8056395114)

AuNPsIntensityConfB <- c(11188.0626151829,10772.5481345214,9846.5363715132)


#FeONP, single exposure

FeONPsArea <- c(3.4470556793  ,4.2657934035, 6.4121840214)

FeONPsAreaSD <- c(1.7132825429,  3.0082987031,      3.920497569)

FeONPsAreaConfA <- c(3.0171114114 , 3.6671293521     ,5.6587024925)

FeONPsAreaConfB <- c(3.8769999473,  4.8644574548,    7.1656655503)

FeONPsIntensity <- c(11609.9125861969 , 10325.9374625671    ,10066.4750261819)

FeONPsIntensitySD <- c(2204.554788687 , 1736.9767232019,    1566.5446158882)

FeONPsIntensityConfA <- c(11056.6846911783,  9980.2718140246       ,9765.4003765232)

FeONPsIntensityConfB <- c(12163.1404812155  ,10671.6031111095,      10367.5496758406)


#AuNP, coexposure

AuNPcArea <- c(1.4811287403,  2.1363252353,  6.1642135964)

AuNPcAreaSD <- c(0.6156055407,  1.100126492, 2.6749622161)

AuNPcAreaConfA <- c(1.3573378152,  1.896746535,      5.6501118568)

AuNPcAreaConfB <- c(1.6049196654,  2.3759039356,     6.6783153359)

AuNPcIntensity <- c(8487.6948839891,  6765.2418404087,      9114.4780307686)

AuNPcIntensitySD <- c(1763.568894204,  1277.0555907495,     1633.8688469273)

AuNPcIntensityConfA <- c(8133.0622330257,  6487.1326221727, 8800.4643182398)

AuNPcIntensityConfB <- c(8842.3275349526,  7043.3510586448, 9428.4917432973)
```

```
#FeONP, coexposure

FeONPcArea <- c(3.6352243739,  4.3600638162, 8.7714426327)

FeONPcAreaSD <- c(1.7519325651,  1.986232463,       4.237195)

FeONPcAreaConfA <- c(3.2829316498,  3.9275144726,    7.9570949357)

FeONPcAreaConfB <- c(3.987517098,  4.7926131598,     9.5857903297)

FeONPcIntensity <- c(9365.5690782627,  8081.4095880043,     9720.9203193921)

FeONPcIntensitySD <- c(1728.8957737482,  1512.8614425605,   1195.9402061447)

FeONPcIntensityConfA <- c(9017.9087778917,  7751.9480390023,       9491.0722353184)

FeONPcIntensityConfB <- c(9713.2293786336 , 8410.8711370063,       9950.7684034658)


time<-c(2,6,24)

ylimitsIntensity <-c(0,15000)

ylimitsArea <-c(0,12)


# Area plots AuNP single

plot(time, AuNPsArea, ylim=ylimitsArea, pch =15)

points(time, AuNPsAreaConfA)

points(time, AuNPsAreaConfB)

points(time,AuNPsArea-AuNPsAreaSD)

points(time,AuNPsArea+AuNPsAreaSD)


# Intensity plot AuNP single

plot(time, AuNPsIntensity, ylim=ylimitsIntensity, pch =15)

points(time, AuNPsIntensityConfA)

points(time, AuNPsIntensityConfB)

points(time,AuNPsIntensity-AuNPsIntensitySD)

points(time,AuNPsIntensity+AuNPsIntensitySD)


# Area plot FeONP single

plot(time, FeONPsArea, ylim=ylimitsArea, pch =15)

points(time, FeONPsAreaConfA)

points(time, FeONPsAreaConfB)

points(time,FeONPsArea-FeONPsAreaSD)

points(time,FeONPsArea+FeONPsAreaSD)


# Intensity plot FeONP single

plot(time, FeONPsIntensity, ylim=ylimitsIntensity, pch =15)
```

```
points(time, FeONPsIntensityConfA)

points(time, FeONPsIntensityConfB)

points(time,FeONPsIntensity-FeONPsIntensitySD)

points(time,FeONPsIntensity+FeONPsIntensitySD)


# Area plots AuNP coexposure

plot(time, AuNPcArea, ylim=ylimitsArea, pch =15)

points(time, AuNPcAreaConfA)

points(time, AuNPcAreaConfB)

points(time,AuNPcArea-AuNPcAreaSD)

points(time,AuNPcArea+AuNPcAreaSD)


# Intensity plot AuNP coexposure

plot(time, AuNPcIntensity, ylim=ylimitsIntensity, pch =15)

points(time, AuNPcIntensityConfA)

points(time, AuNPcIntensityConfB)

points(time,AuNPcIntensity-AuNPcIntensitySD)

points(time,AuNPcIntensity+AuNPcIntensitySD)


# Area plot FeONP coexposure

plot(time, FeONPcArea, ylim=ylimitsArea, pch =15)

points(time, FeONPcAreaConfA)

points(time, FeONPcAreaConfB)

points(time,FeONPcArea-FeONPcAreaSD)

points(time,FeONPcArea+FeONPcAreaSD)


# Intensity plot FeONP coexposure

plot(time, FeONPcIntensity, ylim=ylimitsIntensity, pch =15)

points(time, FeONPcIntensityConfA)

points(time, FeONPcIntensityConfB)

points(time,FeONPcIntensity-FeONPcIntensitySD)

points(time,FeONPcIntensity+FeONPcIntensitySD)


#COLOCALIZATION LYSOSOMES / FeO and AuNP

#===========================================

#DATA: Pearson's colocalization coefficients between particles (AuNP or FeO) and Lysosomes.

#The index 2h, 6h and 26h denotes the incubation time
```

```
# Lysosomes with AuNP single exposure
AuNPsLysMean <- c(0.214599823,0.2352908,0.596951685)
AuNPsLysSD <-c(0.119186217,0.143290654,0.118703123)
AuNPsLysConfA <-c(0.184932505,0.199033928,0.572290427)
AuNPsLysConfB <-c(0.24426714,0.271547672,0.621612944)

# Lysosomes with FeONP single exposure
FeONPsLysMean <- c(0.555122951,0.568125773,0.632516346)
FeONPsLysSD <-c(0.069117153,0.073398605,0.126801009)
FeONPsLysConfA <-c(0.537778162,0.553519143,0.608146425)
FeONPsLysConfB <-c(0.572467739,0.582732403,0.656886268)

# Lysosomes with AuNP Coexposure
AuNPcLysMean <- c(0.253919789,0.274585679,0.565125)
AuNPcLysSD <-c(0.08262379,0.147643709,0.103280256)
AuNPcLysConfA <-c(0.237305132,0.242432751,0.545275539)
AuNPcLysConfB <-c(0.270534447,0.306738607,0.584974461)

# Lysosomes with FeONP Coexposure
FeONPcLysMean <- c(0.448036105,0.464438272,0.568493269)
FeONPcLysSD <-c(0.086417019,0.078248055,0.103119112)
FeONPcLysConfA <-c(0.430658674,0.447397897,0.548674778)
FeONPcLysConfB <-c(0.465413536,0.481478646,0.58831176)

AuNPsLys2h <-
c(0.2458,0.06765,0.3149,0.06765,0.2596,0.4665,0.2865,0.06765,0.3129,0.3438,0.2048,0.2827,0.3094,0
.06765,0.09903,0.3333,0.2021,0.2686,0.3104,0.3524,0.06765,0.121,0.254,0.3027,0.09939,0.1641,0.235
,0.3259,0.1677,0.008109,0.3157,0.1261,0.06765,0.3613,0.2324,0.2435,0.3727,0.1122,0.2191,0.1441,0.
2655,0.3405,0.1623,0.2661,0.1539,0.1085,0.3905,0.3103,-0.0217,0.2808,0.3121,0.1272,0.2174,0.462,-
0.07745,0.2662,0.3054,0.2773,0.06765,0.06765,0.08731,0.1321)
AuNPsLys6h <- c(0.3857,0.2228,-
0.04072,0.04381,0.1495,0.08596,0.2822,0.147,0.1979,0.04953,0.2682,0.2293,0.02922,0.272,0.4004,0.2
058,0.3275,0.2683,0.2334,0.1546,0.187,0.1964,0.06549,0.3947,0.2957,0.3106,0.2586,0.4156,0.4079,0.
3248,0.08742,0.1175,0.2781,0.2641,0.1697,0.0191,0.08921,0.1712,0.2266,0.3268,0.158,0.4595,0.2426,
0.4584,0.3206,0.5741,0.4392,0.1733,-
0.07989,0.2921,0.06255,0.004118,0.2298,0.3481,0.159,0.3781,0.07345,0.4818,0.4414,0.3823)
```

```
AuNPsLys24h <-
c(0.5862,0.6512,0.707,0.6204,0.5655,0.7215,0.6358,0.6257,0.6329,0.6753,0.2929,0.5712,0.7291,0.715
,0.7059,0.678,0.6893,0.5282,0.5997,0.6263,0.6628,0.4228,0.5745,0.7663,0.5209,0.6572,0.5632,0.6191
,0.6707,0.7143,0.5956,0.6438,0.6629,0.4872,0.3439,0.7063,0.5983,0.6093,0.2685,0.6684,0.6515,0.663
,0.632,0.7187,0.5881,0.4777,0.6741,0.5857,0.6939,0.6794,0.605,0.5096,0.3904,0.6754,0.7074,0.6028,
0.6807,0.738,0.5106,0.6758,0.4976,0.6791,0.6719,0.5549,0.5767,0.5963,0.567,0.4986,0.6507,0.3588,0
.6315,0.2469,0.7039,0.5638,0.5179,0.7355,0.4283,0.6501,0.6667,0.6642,0.7586,0.4998,0.1973,0.3543,
0.6549,0.6807,0.5264,0.5738,0.6496)

FeONPsLys2h <-
c(0.4925,0.4888,0.4882,0.5546,0.6065,0.5028,0.5531,0.5728,0.6121,0.4938,0.494,0.6047,0.6699,0.593
7,0.6457,0.5559,0.5706,0.547,0.6602,0.544,0.5639,0.5883,0.56,0.5581,0.5567,0.5577,0.4389,0.5368,0
.6531,0.3071,0.5481,0.4529,0.4312,0.5551,0.5201,0.5374,0.5831,0.5887,0.5583,0.5481,0.5459,0.5746,
0.5644,0.5918,0.6056,0.6383,0.3965,0.6493,0.6358,0.6055,0.6731,0.4924,0.5349,0.5059,0.6637,0.619,
0.4585,0.578,0.4988,0.5493,0.5867)
FeONPsLys6h <-
c(0.5564,0.7223,0.6314,0.5208,0.6221,0.4668,0.6191,0.6007,0.6019,0.5974,0.5186,0.6021,0.6266,0.57
66,0.5513,0.5545,0.6175,0.6969,0.6595,0.5743,0.5589,0.6065,0.645,0.5755,0.6647,0.6007,0.4133,0.61
12,0.543,0.6064,0.6691,0.6745,0.539,0.5653,0.618,0.5536,0.6433,0.4982,0.5462,0.6142,0.4814,0.532,
0.6555,0.5058,0.5899,0.5458,0.525,0.6667,0.5853,0.5563,0.6537,0.5705,0.6225,0.5512,0.6534,0.5133,
0.5933,0.3546,0.5994,0.627,0.3698,0.5397,0.5701,0.6773,0.6178,0.564,0.5221,0.512,0.5758,0.5412,0.
6616,0.5512,0.5263,0.5715,0.5642,0.5537,0.5676,0.5143,0.5232,0.5361,0.5235,0.6024,0.5419,0.4651,0
.5034,0.5574,0.6058,0.5396,0.6072,0.6152,0.5918,0.6432,0.2361,0.5033,0.5108,0.5085,0.4475)
FeONPsLys24h <-
c(0.619,0.7026,0.6262,0.7501,0.673,0.6309,0.3867,0.7127,0.717,0.7261,0.424,0.7446,0.7463,0.771,0.
6433,0.3121,0.5641,0.5477,0.6638,0.6815,0.7725,0.6642,0.7788,0.7072,0.7327,0.4792,0.7845,0.7112,0
.3583,0.5131,0.741,0.8005,0.6212,0.6334,0.6989,0.668,0.6161,0.7584,0.7427,0.6098,0.5962,0.6418,0.
32,0.7567,0.6419,0.8235,0.7091,0.3386,0.5926,0.6492,0.6406,0.6922,0.6677,0.6841,0.5592,0.5335,0.5
005,0.6385,0.8073,0.7316,0.723,0.6196,0.3576,0.6753,0.5937,0.6126,0.699,0.5689,0.6032,0.7137,0.37
45,0.7926,0.6751,0.6519,0.6644,0.6278,0.7528,0.5523,0.5836,0.635,0.6448,0.6622,0.5473,0.3485,0.65
78,0.6895,0.7693,0.5602,0.7778,0.7316,0.5117,0.6709,0.7806,0.7455,0.6901,0.6787,0.5945,0.1991,0.3
427,0.7507,0.473,0.5982,0.6121,0.7091)

AuNPcLys2h <-
c(0.1929,0.1532,0.2832,0.3169,0.2213,0.308,0.2788,0.2806,0.05016,0.343,0.2618,0.3171,0.3473,0.292
6,0.2242,0.2573,0.3808,0.198,0.2377,0.2117,0.1198,0.1234,0.2328,0.08847,0.278,0.185,0.2238,0.2783
,0.2223,0.4024,0.2629,0.3788,0.3467,0.1979,0.2847,0.2235,0.2954,0.3825,0.2018,0.2392,0.2992,0.288
```

8,0.2654,0.4071,0.2685,0.2385,0.2618,0.2874,0.2585,0.4125,0.2111,0.3159,0.2438,0.4049,0.2517,0.29
62,0.4222,0.2521,0.2473,0.2484,0.05825,0.2259,0.2171,0.3122,0.2744,0.3612,0.109,0.3055,-
01147,0.1884,0.3067,0.1955,0.1823,0.2103,0.07067,0.3446,0.1981,0.2887,0.1863,0.2716,0.2227,0.1816
,0.2682,0.3394,0.2445,0.2418,0.1084,0.2566,0.265,0.3626,0.32,0.1955,0.2984,0.2369,0.28)
AuNPcLys6h <- c(0.4045,0.1559,-
0.1569,0.3929,0.4396,0.2402,0.3574,0.01906,0.3787,0.1962,0.1043,0.1811,0.3412,0.3283,0.3886,0.085
28,0.3239,0.04809,0.3316,0.1236,0.1406,0.3245,0.3697,0.4199,0.3277,0.2659,0.2421,0.2839,0.4117,0.
3532,0.3136,0.288,0.3174,0.2544,0.427,0.4427,0.4549,0.2565,0.292,0.215,0.2281,0.2746,0.4438,0.342
4,0.3835,-
0.02226,0.3352,0.1742,0.335,0.3047,0.5488,0.3059,0.3243,0.0787,0.118,0.1828,0.2864,0.07245,0.3063
,0.4774,-0.08958,0.3702,0.3703,0.3276,-
0.03693,0.131,0.522,0.1324,0.574,0.2633,0.4575,0.2079,0.4048,0.4349,0.01399,0.2784,0.366,0.06074,
0.3507,0.1829,0.3348)
AuNPcLys24h <-
c(0.519,0.5719,0.6265,0.4946,0.6986,0.6401,0.3992,0.4804,0.3766,0.6436,0.5495,0.46,0.6681,0.643,0
.5981,0.5575,0.6262,0.4901,0.5561,0.5556,0.6878,0.6963,0.6008,0.3974,0.6439,0.5572,0.5019,0.5787,
0.2924,0.2365,0.6167,0.4791,0.5426,0.3958,0.4198,0.5933,0.4465,0.5823,0.7538,0.6532,0.4318,0.6408
,0.6871,0.6067,0.4207,0.6782,0.5925,0.3477,0.6615,0.6304,0.5815,0.4194,0.5203,0.5537,0.5382,0.530
7,0.6819,0.6182,0.5757,0.5122,0.4797,0.6447,0.6714,0.6455,0.7181,0.548,0.6224,0.6957,0.673,0.4276
,0.3708,0.704,0.5666,0.6657,0.6799,0.6349,0.5294,0.6018,0.5952,0.4261,0.6198,0.4968,0.4121,0.5689
,0.6774,0.6334,0.5684,0.6661,0.5036,0.6313,0.3779,0.4763,0.5466,0.6605,0.624,0.5865,0.5873,0.6735
,0.6667,0.6179,0.4591,0.6536,0.6358,0.4691)

FeONPcLys2h <-
c(0.5557,0.4233,0.4423,0.4349,0.5439,0.4755,0.4397,0.4766,0.3427,0.3447,0.4735,0.5252,0.5099,0.51
23,0.4644,0.4612,0.5423,0.411,0.3063,0.573,0.3481,0.4271,0.5483,0.5439,0.5466,0.4759,0.3802,0.303
1,0.5115,0.477,0.3239,0.3713,0.3627,0.388,0.4492,0.388,0.5594,0.4767,0.5262,0.5398,0.4293,0.4762,
0.3568,0.4361,0.3588,0.5146,0.4367,0.4708,0.4382,0.4597,0.4585,0.3927,0.5125,-
0.01707,0.3926,0.5197,0.4407,0.5426,0.4485,0.4661,0.4174,0.5401,0.369,0.3563,0.5262,0.4247,0.5109
,0.4804,0.426,0.4597,0.5089,0.555,0.4381,0.4788,0.51,0.2926,0.532,0.3045,0.3683,0.4829,0.3593,0.5
413,0.5127,0.4479,0.4699,0.5435,0.4341,0.4498,0.4651,0.4078,0.6165,0.3415,0.3503,0.4404,0.4622)
FeONPcLys6h <-
c(0.6101,0.3732,0.4141,0.5602,0.5842,0.5515,0.4319,0.4509,0.4869,0.472,0.3807,0.3508,0.3739,0.553
1,0.5656,0.3627,0.42,0.4342,0.4645,0.3357,0.4461,0.493,0.3838,0.5237,0.5052,0.4655,0.3704,0.4867,
0.4934,0.426,0.4796,0.5152,0.5612,0.4027,0.4769,0.4026,0.3812,0.4731,0.4339,0.4945,0.5746,0.6191,
0.4174,0.4406,0.5093,0.43,0.4117,0.5204,0.4645,0.5794,0.4984,0.4946,0.4265,0.4486,0.4803,0.1887,0

```
.4514,0.4622,0.4165,0.5074,0.4828,0.5283,0.4424,0.4235,0.4664,0.4748,0.4231,0.676,0.594,0.4505,0.
5688,0.3575,0.5471,0.4866,0.4353,0.4307,0.3436,0.5895,0.4109,0.3727,0.3825)
FeONPcLys24h                                                                                    <-
c(0.6259,0.5601,0.4892,0.6685,0.5186,0.6886,0.5795,0.6139,0.5444,0.3751,0.679,0.4873,0.7863,0.484
4,0.7245,0.5526,0.5754,0.4748,0.4952,0.6764,0.6187,0.4812,0.4712,0.4259,0.5427,0.5473,0.6074,0.72
,0.4381,0.663,0.7291,0.6664,0.5345,0.7479,0.4509,0.5938,0.4206,0.681,0.718,0.5957,0.4716,0.6324,0
.4695,0.5131,0.7148,0.5004,0.4614,0.5274,0.5002,0.4941,0.5437,0.653,0.6357,0.5847,0.4414,0.6441,0
.4751,0.6099,0.4936,0.6661,0.701,0.7613,0.6645,0.5949,0.6927,0.4507,0.4478,0.4472,0.6867,0.6482,0
.6949,0.4707,0.4664,0.5293,0.7049,0.4599,0.2072,0.6305,0.5258,0.5802,0.6701,0.4762,0.5456,0.58,0.
5304,0.6228,0.4822,0.5277,0.5107,0.6731,0.5545,0.5061,0.5407,0.5282,0.469,0.4522,0.5663,0.6181,0.
4386,0.6904,0.4443,0.6405,0.6288,0.7767)

# Students's t test
# Difference between exposure time (2 and 6 hours)
t.test(AuNPsLys2h, AuNPsLys6h)
t.test(FeONPsLys2h, FeONPsLys6h)
t.test(AuNPcLys2h, AuNPcLys6h)
t.test(FeONPcLys2h, FeONPcLys6h)

# Difference between exposure time (6 and 24 hours)
t.test(AuNPsLys24h, AuNPsLys6h)
t.test(FeONPsLys6h, FeONPsLys24h)
t.test(AuNPcLys24h, AuNPcLys6h)
t.test(FeONPcLys24h, FeONPcLys6h)

# Difference between NP type
t.test(AuNPsLys2h, FeONPsLys2h)
t.test(AuNPsLys6h, FeONPsLys6h)
t.test(AuNPsLys24h, FeONPsLys24h)
t.test(AuNPcLys2h, FeONPcLys2h)
t.test(AuNPcLys6h, FeONPcLys6h)
t.test(AuNPcLys24h, FeONPcLys24h)

# Difference between single and coexposure
t.test(AuNPsLys2h, AuNPcLys2h)
t.test(AuNPsLys6h, AuNPcLys6h)
t.test(AuNPsLys24h, AuNPcLys24h)
```

```
t.test(FeONPsLys2h, FeONPcLys2h)

t.test(FeONPsLys6h, FeONPcLys6h)

t.test(FeONPsLys24h, FeONPcLys24h)


#INTENSITY & NUMBER OF PIXELS

#AuNPxyh: Particle: AuNP or FeONP, x=(s) or (c)oexposure, y=2,6 or 24 hours

AuNPs2hNumber                                                                                    <-
c(1101,802,3507,409,950,842,559,1239,963,1094,2111,940,2040,2238,2379,3013,1851,2702,369,1844,948
,317,4558,1499,2151,615,4528,1436,893,1169,1719,1306,1640,4186,2042,1731,3637,2358,3080,2458,2227
,1114,1196,1627,1248,1566,1717,1331,1311,1419,1471,788,951,2298,1942,605,1794,2331,1429,568,954,2
532)

AuNPs6hNumber                                                                                    <-
c(1682,2164,1595,542,2480,430,834,3474,1137,2602,1425,1944,888,931,2305,1453,1101,1269,1351,2017,
4531,717,1311,1077,2167,1038,1793,1269,2258,2395,1641,2085,1840,1542,2342,1456,2379,941,1896,9358
,1782,1671,3296,1476,1842,4023,3206,5185,5904,4641,5362,4205,10140,2978,4423,2139,4254,585,752,11
72)

AuNPs24hNumber                                                                                   <-
c(7676,3060,7108,2141,980,1430,7221,8918,8778,8754,12820,1605,9815,14027,18817,10209,10484,7045,5
251,12901,8454,10108,6407,12357,12882,4714,11527,10113,12752,5771,6685,5283,6035,4238,5280,11627,
3332,6409,15566,13427,9132,6869,9997,8399,5329,11034,12510,9156,6170,8935,11901,5678,5805,6271,70
93,201,11920,8392,10079,9006,9601,6760,8982,7452,7624,7123,2715,10947,10410,11771,4660,7617,20982
,9173,7158,7423,5174,720,4955,5532,11636,6417,7602,7479,8336,13186,11958,9588,7319)


AuNPs2hIntensity                                                                                 <-
c(7235.653846,8238.717528,9545.196398,6275.9475,7142.008502,6793.226891,4524.943636,9664.113821,8
225.115304,12033.68111,10064.77688,6677.881847,6637.461841,8092.90489,6141.277215,11537.80326,141
65.41042,11934.80505,12813.78611,13859.54114,13345.01917,21017.60065,12148.08529,12879.26443,7639
.301587,13068.34488,10188.1237,11611.03714,10547.95362,9586.793966,11378.52164,10677.79183,10369.
50582,12289.46229,11941.4181,15798.9669,8326.496968,6716.398042,9150.405405,13837.52593,11742.331
38,7316.242534,5877.47936,7829.749073,9293.920097,7681.2158,7295.912178,17666.25946,17181.89247,1
4534.16383,11977.78591,8299.686778,13746.75902,16380.57886,12108.34247,6289.332215,11347.62857,13
311.4509,12533.53662,4649.878354,5831.86455,10471.96195)

AuNPs6hIntensity                                                                                 <-
c(14858.1835,9060.442227,11474.00252,10651.4878,11461.64185,8505.456057,14014.87152,12750.44906,1
2524.85461,13913.28808,13497.07062,8883.197933,11602.18316,8990.944685,15579.75,10236.29501,11895
.5467,9664.156349,10656.36736,9668.178287,9459.578726,8409.336158,8247.463134,11704.60019,12321.7
0343,12648.13411,11598.70796,10017.13492,10197.41574,10303.55574,10214.91605,11818.16329,7744.984
```

```
162,10479.14547,9625.66781,11065.93504,11698.75781,13601.68562,10956.62533,8872.189325,6668.96164
7,10994.67509,7055.09279,7721.486708,11316.33442,7546.567265,7298.51736,7037.454598,5871.9581,507
0.042314,7998.775453,4456.098904,5161.082124,5639.962614,5005.548482,9342.576995,5393.793875,1232
6.86979,17637.28668,8444.235598)
AuNPs24hIntensity                                                                              <-
c(12780.52054,12289.95084,9982.481195,10549.68058,9951.505664,11573.04785,6436.408625,8048.053317
,10569.36618,8184.00526,9460.462103,10469.91604,9597.720783,8274.984092,9078.605647,6911.172549,7
762.323628,8623.984935,7150.379435,6995.405678,7839.623919,8072.198237,11097.44389,8507.077907,83
48.436806,8416.283528,7873.349627,8240.61154,9561.439378,12500.416,12382.61055,6395.647895,7713.5
32194,13919.69142,7190.572187,8501.953606,6342.78062,7716.288281,9531.406569,8328.363691,6613.195
879,8447.092711,6212.352924,6330.320024,10805.85508,7773.332063,10737.11639,10913.4033,11845.2739
8,10590.46527,6076.506223,6032.107426,6180.616287,8748.530342,9369.163749,16154.08854,12268.19738
,16951.17953,13452.59176,11018.59931,9257.910655,11879.00074,10899.82392,9949.024049,9564.833487,
9757.647456,12914.59904,11798.65195,12391.10682,12475.23678,7613.934208,15407.25,14359.58656,1092
4.27335,12545.5114,11198.94969,11939.72662,7901.21097,8522.773554,11322.12892,15423.11955,14664.9
8112,18139.76557,16547.87041,12465.51411,13791.55961,12735.81806,12040.49441,14032.2316)

FeONPs2hNumber                                                                                 <-
c(6544,6339,9793,7970,3010,3820,6089,2116,1902,4356,2584,3500,5191,4514,6127,5236,2459,5660,5261,
2686,4698,5470,2229,5370,7017,5460,9199,8884,7376,4365,7826,3329,5397,4013,2662,10078,7828,8826,6
194,4223,4486,8629,9306,11809,7845,4800,4559,6457,4191,8413,5454,6151,6926,12989,4628,3142,4897,4
510,4594,5792,5125)
FeONPs6hNumber                                                                                 <-
c(3140,5467,11204,6010,5132,6517,5497,2253,4327,3667,4642,9621,4227,2945,2211,8022,7811,4211,3695
,4622,7957,5538,5050,5133,1245,5607,2763,3632,5354,2935,4549,4513,4868,2661,6102,3211,3876,1487,2
397,1146,6887,19360,5130,2698,3507,5430,2947,3696,3151,1196,3182,7146,2691,10965,4748,3327,3704,6
738,3755,5301,2951,6064,5117,6274,14093,13075,3248,26704,15645,11167,18823,15631,7073,4730,6952,9
491,5680,3673,10703,13300,16655,10024,12913,8154,7125,11160,12580,9590,4114,10152,8628,10456,9152
,12298,5010,9127,3738)
FeONPs24hNumber                                                                                <-
c(2028,3650,19709,19068,17415,30746,23090,400,765,1437,3401,4273,2759,2951,6844,3898,763,3481,168
4,2706,2044,1303,3991,13289,7756,6459,2059,4086,1460,4182,3077,7192,1777,5141,2192,2576,2600,3597
,4886,1727,3231,4396,14561,6392,5991,3344,2141,6712,1224,2158,2711,2474,2518,2427,6839,807,911,10
74,17520,8121,3807,6580,3348,19947,17634,13693,13553,14748,13265,12271,13739,1831,10915,17609,153
75,23279,14867,20877,21411,26305,32216,23924,2385,39003,28918,47616,51542,28006,27232,26236,12023
,13723,13257,3625,11363,18781,19963,7616,18845,19408,22100,19375,20113,22696)
```

```
FeONPs2hIntensity                                                                                    <-
c(10182.82999,8471.551185,11484.69982,12673.57706,10005.63079,8296.846235,9199.304441,10138.66682
,7556.057052,10686.87923,10350.5266,7469.089373,14241.89579,8265.021088,10510.63223,12700.25464,1
0667.31265,12594.07503,16208.92022,11078.51289,14738.68586,14944.81743,7145.312613,10838.66144,11
986.31892,10683.66061,10463.74897,10368.25701,12229.06488,9424.608356,12346.10618,6000.039157,853
0.093356,5263.909341,9563.003015,9065.735525,10954.43995,16112.14291,12311.35327,15005.88277,1655
0.79048,16220.85058,13210.21372,14618.02941,5828.827463,15541.24358,11822.55341,16808.54653,18190
.66714,15106.78296,14527.56933,10000.08336,13267.27454,13354.60786,16077.12427,11972.0316,14010.1
7042,9608.321928,12168.64297,8443.920629,10118.29085)
FeONPs6hIntensity                                                                                    <-
c(8402.778984,8554.877611,8295.95498,9029.052158,11153.34726,11159.36816,12161.22103,7942.074866,
9538.24641,10191.21897,11333.97345,7695.708073,11825.37387,11755.51396,7594.331063,12800.64682,13
864.25699,12973.2694,11080.89881,10237.66746,13466.39582,13638.28414,15564.75699,8814.314403,1068
9.91909,8561.927831,13258.97277,10307.84516,12932.21759,12445.03281,9862.772467,9544.587034,10157
.55814,10750.77489,9853.90645,6769.201749,8203.615206,9305.771313,8063.582077,7449.741425,8987.99
3167,10140.86915,11384.99746,7993.703235,5710.741281,10820.35326,7757.87951,8694.487659,9106.8239
97,7268.211457,9200.674125,14905.70057,11382.48471,12103.70518,7258.784132,9213.485232,10729.5732
1,12672.47347,12079.78457,9033.445578,12580.22162,13950.77242,13246.78543,15935.97047,12359.90308
,11211.97245,7946.842235,6727.761379,8207.535367,11856.62547,7209.592378,7233.520804,11442.19182,
13216.01546,14738.07634,13762.98049,7182.505026,6639.878002,8033.998691,10663.52818,8285.772798,8
430.389516,10254.99264,9470.755064,9362.911467,12191.92037,5694.250577,5775.736458,6040.521315,92
67.440895,9922.136675,6570.057816,9337.998469,9654.197738,8837.95061,8246.583023,9954.919281)
FeONPs24hIntensity                                                                                   <-
c(10738.80535,8745.289481,15458.76096,17773.13694,15597.39239,13597.88346,13829.96092,9797.769821
,10359.62434,8112.052521,9530.293337,9413.685272,10387.35164,8771.701903,10880.59049,8830.369504,
8998.399204,12404.44153,12557.2203,11974.16426,9498.62801,8921.304482,9368.218232,8028.14375,6921
.060152,8236.613023,8413.703902,12143.68433,8717.644383,8292.279895,7670.21219,8635.557566,7537.7
80543,10569.19953,11398.27256,9572.480717,8375.09147,9468.410814,8590.173467,9425.25844,11812.511
79,11758.61477,9424.408741,9700.995457,9207.096122,7662.856072,9839.787523,9178.517679,10113.9497
9,10827.81666,7475.210215,10779.48195,10545.29454,7611.04756,8486.331918,10025.58271,9790.822616,
9984.849765,11249.05871,10620.74063,13951.4634,6532.401918,10529.15244,11340.30179,12502.62411,13
042.99817,11329.53832,11725.91241,11018.79707,14470.13888,13119.05025,9510.421515,14819.06648,124
30.4483,15262.27366,12335.54998,15362.98311,14478.24085,11008.29946,11486.90082,14358.34974,17244
.16881,7729.872896,14038.75742,15358.30651,15284.47398,11349.2303,14327.4978,13465.17162,14399.50
864,14633.7885,14452.13811,14195.58764,8255.551991,18070.16611,16547.76135,18771.9545,14897.82345
,12909.91569,13728.90412,14098.98941,15587.53377,16645.56541,16382.40671)
```

```
AuNPc2hNumber <-
c(2287,1239,1942,1663,3893,1672,1863,2013,1545,3870,2067,2441,2207,3679,3918,2210,3792,2997,1460,
1910,1215,2256,1663,1242,1552,1280,2016,1658,1835,1620,2478,1851,1455,3509,956,2157,4316,2287,341
1,2573,1871,4272,2496,2110,3708,1121,1679,2184,2281,1392,4022,1501,2282,2167,3890,2315,3142,3066,
2064,3391,3731,2613,2841,1106,1557,1721,3776,1550,1250,1385,2067,2042,1934,1791,2768,1828,3367,29
55,3103,1619,1487,1802,1755,2206,2346,4477,2866,1257,2287,1791,602,1031,996,871,1979)
AuNPc6hNumber <-
c(3064,1675,1078,1489,2497,1842,2888,1064,1762,2986,1177,2377,3218,2672,7113,4671,1404,4324,1462,
2144,569,1715,1041,827,2212,3627,3837,2262,3554,2479,1175,4116,1297,481,1827,2677,1757,2366,6995,
6465,4236,4498,3016,4468,1298,2424,4130,4592,4514,2373,1158,3980,2236,7448,4333,816,3874,8537,379
2,6074,3846,4061,3894,4055,2522,5047,1913,2672,2556,2254,4029,2760,4406,4597,2203,3332,6579,4184,
1920,3403,3440)
AuNPc24hNumber <-
c(13045,18742,9222,15850,12812,8773,16335,15364,15172,16014,12379,19722,17049,16700,22601,14603,1
5125,2036,2737,2302,959,1591,4671,3729,3692,4933,1914,20279,4060,2286,2908,2145,3201,2295,7245,24
32,2473,4402,22135,1089,1333,7530,12807,17843,7422,9615,8358,11195,9366,15651,9678,11283,13481,13
504,13068,10761,17629,7699,5399,13934,17958,7383,13746,6575,10293,11445,19333,9667,13484,5939,548
9,15001,10489,8947,13774,11028,10713,12281,3164,3846,630,6770,13787,5188,9222,11479,10125,11939,1
1174,13305,10736,10858,8866,15420,10434,10904,16176,12658,12038,7246,8277,10450,6837,11188)
AuNPc2hIntensity <-
c(9147.517559,6764.769919,6711.639938,8261.677751,10036.65165,9552.80457,7652.158037,5898.841317,
6041.824219,5647.683243,5779.651603,7565.755757,5530.480892,7847.575204,8967.965464,7622.26806,64
89.743325,8878.794511,8710.658167,6750.636507,6850.592869,9149.048509,8261.677751,10534.78994,120
13.19961,13025.74115,13799.21126,9970.252274,12267.11884,11036.30602,7192.324423,6828.910966,7190
.058091,8147.640571,6569.206969,7279.182495,6870.43023,6297.852941,7336.570841,5849.302262,6915.0
26316,6205.216045,9308.669079,6889.003332,9145.23655,5738.103417,6972.653293,7275.274023,7696.227
113,6993.11786,5335.138301,5494.006032,6794.072591,6604.847081,7911.131925,7676.143539,6761.82700
3,7129.10664,8494.546472,7373.166765,8030.632724,7531.011521,6590.209393,7394.498633,6612.638243,
6927.397196,6475.561189,12013.34393,10399.2361,12766.80305,13572.21186,10730.45647,11991.1226,106
14.6633,10984.45379,10290.90269,13033.03514,8701.454515,9305.214286,9458.26087,9057.857916,9937.5
0251,9528.668958,11579.97724,11217.06975,9799.093107,10886.89114,9970.52484,9111.748025,9971.0359
15,7769.107926,11516.00196,7903.906788,7937.324826,7680.166497)
AuNPc6hIntensity <-
c(7388.479542,7702.510804,5627.830683,6529.290541,6177.028537,9006.697763,7588.300452,4740.090047
,7295.476897,7166.517971,7309.694349,7669.30152,6326.462138,7427.376643,7003.900479,6458.047405,5
377.936201,6522.765933,7031.417068,7963.104918,4923.782143,6178.148886,10194.52907,5919.413203,53
85.641398,7817.454671,6821.01698,7761.588105,7240.08237,6256.365587,7388.61235,6912.720721,7658.8
```

```
52484,4406.887712,9523.406491,6123.090705,5826.556636,6617.441663,6154.514314,6567.053903,6654.74
8285,5699.552016,5482.65148,6301.426105,8283.031808,5807.890269,5287.902451,5460.700196,5565.6290
79,4525.193739,4189.56832,5698.534878,6049.044454,7626.437693,8440.546253,7415.444857,6664.747477
,9923.004925,6994.773196,7249.266777,6705.919468,6402.46693,8026.664865,7770.585517,6978.25189,67
23.960103,6384.636029,5957.911378,5393.464468,4843.483296,5304.050995,6877.065794,7487.27405,7771
.058631,6439.021422,7733.242552,7811.147641,6835.937964,7184.789116,7147.216559,8896.956864)
AuNPc24hIntensity                                                                                <-
c(7620.466094,9061.227993,7545.125583,8631.241588,8341.556745,8564.733455,6811.285312,8547.115077
,9291.365759,8175.973821,7974.539935,9901.58885,9310.117195,10336.02379,11466.32879,10239.06516,9
309.77666,7485.669462,7734.755499,8558.299171,8403.908421,8873.003793,10162.95646,8330.281183,889
9.323649,9036.52234,8439.545407,9009.864726,9669.858307,9462.30874,11534.92549,8894.158708,8379.5
06579,8504.322835,11135.572,8773.877425,9413.986607,10573.79695,9825.308732,8063.102778,8462.2371
6,7593.366175,8889.903969,8335.896826,6777.855794,8554.027171,7700.206971,10322.83408,8336.677461
,10206.83308,7466.420623,7031.687334,9188.992726,8665.306113,6844.391914,9698.099609,7590.022815,
6880.246814,6322.388868,8587.016014,10125.35896,8055.800651,10092.05831,7664.367195,7762.374271,9
559.371021,8926.482302,8044.080969,10494.2144,10226.38347,7783.99927,8235.889741,6953.556584,7181
.254419,7286.424482,8635.302931,7894.756446,8012.774038,11816.55531,13100.90566,13132.30918,9351.
735246,9909.989766,9476.252751,8407.193531,6978.78823,9933.295967,11248.67997,12773.71626,11773.1
9006,14496.71082,13651.05088,12255.3246,8691.572059,15063.37688,12593.17696,12299.05975,10839.475
06,6478.739047,6183.863479,5482.454765,7706.93401,8086.604716,9493.51221)

FeONPc2hNumber                                                                                   <-
c(6311,3883,7017,4181,7750,6569,3543,4587,3431,10295,4965,4954,4713,3416,9494,5237,6294,8033,4058
,6863,3798,6098,4181,7829,1944,3035,4086,4387,5619,2576,4416,4815,3103,3420,5272,5483,6911,6043,6
893,5974,8279,10087,3973,5584,9475,9278,8614,4553,8173,11422,10370,2897,6727,4679,9365,5238,7436,
6531,5127,5141,15130,3584,3771,3314,4159,5506,6291,1944,7829,3366,4158,5105,5721,2718,8716,3177,5
338,8025,6185,1619,2660,3875,4138,5078,3743,9579,7216,3694,6312,2645,3546,2314,3049,2939,3649)
FeONPc6hNumber                                                                                   <-
c(6670,8205,4394,2463,8327,3209,7260,9784,10457,7229,4498,6136,8874,5735,12760,6829,7381,3727,170
2,2327,2251,4676,3338,5796,6041,9826,3559,6014,4877,4234,3815,17599,2834,1662,7964,8098,3377,4387
,9294,17642,6235,5743,1982,5738,2828,3672,5295,10908,4617,7050,2070,10156,2341,12057,6281,4515,45
70,11614,8087,10784,7587,7758,6079,4864,3558,7177,3888,7280,4243,3632,6612,7243,11025,11495,2695,
6357,10799,6550,2953,7349,6753)
FeONPc24hNumber                                                                                  <-
c(18789,24822,18411,27433,27824,10908,27607,22699,17717,17004,18150,22782,19483,17990,31720,16330
,19834,2568,1252,548,1892,2521,6986,3975,3350,4747,1996,24215,5407,1471,2416,2234,2867,2336,6318,
3550,3108,6324,23846,1139,1804,16221,15465,24110,12381,12829,13455,13580,9603,21916,12045,20426,1
```

```
8552,21390,25662,21150,24301,10989,8172,18159,27889,9219,17945,11805,10089,9465,23840,24046,14250
,8535,6093,17751,11557,8810,17352,14004,7860,11218,11232,12591,15567,18193,15154,15564,11526,1471
8,12863,10841,17957,23808,16462,13430,11486,19385,22036,22808,23845,21703,19460,12362,11728,17585
,21772,15647)
FeONPc2hIntensity                                                                              <-
c(8353.001904,7970.688436,7803.608162,10234.93696,10301.11536,9049.804573,8719.105829,7897.320446
,7760.968147,6401.45596,5969.274617,9930.934884,7086.672194,9954.022307,11539.89025,9900.46404,73
83.692283,9920.777044,10281.9064,7822.177706,7995.375561,8385.531286,10234.93696,10994.35563,1730
3.19328,11227.37607,13922.88202,9839.204431,11601.38645,12189.92248,10075.18788,7841.431336,9501.
32159,9943.555262,7996.699411,8322.653818,9648.817734,7357.785549,9824.451191,5977.580386,7534.14
8368,7289.162631,12453.08199,8578.418475,11553.76558,6749.633833,7869.497734,9639.127641,8512.276
458,8602.436958,6579.350256,7426.269044,9313.940607,9927.092934,11204.28581,9902.14018,8861.32503
,8795.429163,13735.27218,11469.5567,9462.383639,10741.22182,9530.791866,7579.718003,8688.027229,8
378.316172,7382.607927,17303.19328,10994.35563,11232.82157,13808.78284,10401.56927,11576.85924,12
080.05168,14609.35741,12849.59091,13321.0456,9926.784681,16732.51004,12888.06832,11088.90381,1318
8.60838,15205.93776,12053.2103,14935.73219,11769.84525,12055.00194,10294.32456,8351.693479,11638.
20068,8818.669211,12366.19783,6932.656579,7344.311263,8291.890385)
FeONPc6hIntensity                                                                              <-
c(9446.05855,10074.36567,6884.105359,8701.363896,7359.307165,10992.34281,9153.200524,8272.93913,7
925.962098,8107.019529,9220.954778,8090.032642,7523.379808,9834.126441,8270.490785,8153.884604,62
38.724769,7553.79801,7795.535735,10073.26575,6120.189117,6259.792158,11116.22619,6279.616727,7582
.050895,8517.02567,9492.675211,8716.078934,11032.90941,8421.380118,7994.110352,8223.703297,9219.8
67965,6778.268603,11401.52847,7185.727655,6726.898159,7030.157606,7440.419386,6904.440197,7287.77
5618,6202.14632,6527.17334,8132.731716,11397.42284,7136.521704,5305.385925,5991.537664,6962.03450
5,6225.405766,4874.576419,6985.316153,6623.721698,10457.17289,10964.66964,8285.405903,9052.873054
,11941.00672,7604.552612,7737.9458,6816.434283,6421.659698,8713.978583,8685.69104,7032.826712,771
1.088588,7558.755865,6015.403246,5883.881908,6071.147392,5555.664849,9348.749378,8702.864107,8711
.423037,6657.954207,8502.400599,10782.08165,6544.222137,7204.673913,9145.604768,11380.26157)
FeONPc24hIntensity                                                                             <-
c(8192.223908,10450.22827,8898.348549,11117.4371,9847.162394,10600.87237,8817.058881,9641.432437,
9811.216117,8473.82283,10785.24315,10863.10723,9869.971449,10622.83955,14079.60619,11414.49985,10
324.05261,6935.766315,7458.123089,8424.19666,9895.538502,8687.593153,10513.16368,10207.0996,9900.
861419,9154.174124,9428.649723,9698.112369,10419.11856,10762.16211,12852.2231,9257.692135,10535.9
2022,9369.515685,11935.4278,10413.28523,10007.63311,11426.47348,10691.15723,8687.858407,9947.2367
69,8690.543116,9979.575375,9405.65296,7074.293,9391.298908,8647.709058,11162.68735,9071.641026,10
976.42968,8593.416334,8237.981094,11014.89263,9239.044105,8054.121389,10562.21442,8378.671785,764
7.97623,7120.174201,9447.163416,11275.65599,8307.812378,11133.1143,7838.059681,8400.071329,11469.
```

```
15546,10002.60589,8576.667471,11517.28432,11573.68754,7929.486358,8573.425826,7918.265587,7738.74
4802,7511.83636,9067.631583,9109.660935,9265.074226,10346.47973,11740.15014,9954.172966,9523.5918
94,11111.414,10372.45336,9268.900148,8137.390373,12285.4702,14028.83438,13466.57594,12556.29732,1
5070.03787,13950.6621,12852.25669,9222.104717,15919.89758,13439.23918,13318.27941,11581.68019,734
7.612771,7799.160123,7387.323577,9324.727071,10133.66209,11586.09074)


t.test(AuNPs24hIntensity, AuNPs6hIntensity)
t.test(FeONPs24hNumber, FeONPs6hNumber)
t.test(FeONPs2hIntensity, FeONPs6hIntensity)
t.test(AuNPc24hNumber, AuNPc6hNumber)
t.test(AuNPc2hIntensity, AuNPc6hIntensity)
t.test(FeONPc24hNumber, FeONPc6hNumber)

time<-c(2,6,24)
ylimitsIntensity <-c(0,1)
ylimitsArea <-c(0,1)

#PLOTS
# AuNPs Lysosome Pearsons
plot(time, AuNPsLysMean, ylim=ylimitsArea, pch =15)
points(time, AuNPsLysConfA, pch=3)
points(time, AuNPsLysConfB, pch=3)
points(time,AuNPsLysMean-AuNPsLysSD, pch=3)
points(time,AuNPsLysMean+AuNPsLysSD, pch=3)

# FeONPs Lysosome Pearsons
plot(time, FeONPsLysMean, ylim=ylimitsArea, pch =15)
points(time, FeONPsLysConfA, pch=3)
points(time, FeONPsLysConfB, pch=3)
points(time,FeONPsLysMean-FeONPsLysSD, pch=3)
points(time,FeONPsLysMean+FeONPsLysSD, pch=3)

# COEXPSOURE
# AuNPs Lysosome Pearsons
plot(time, AuNPcLysMean, ylim=ylimitsArea, pch =15)
points(time, AuNPcLysConfA, pch=3)
```

```
points(time, AuNPcLysConfB, pch=3)

points(time,AuNPcLysMean-AuNPcLysSD, pch=3)

points(time,AuNPcLysMean+AuNPcLysSD, pch=3)


# FeONPs Lysosome Pearsons

plot(time, FeONPcLysMean, ylim=ylimitsArea, pch =15)

points(time, FeONPcLysConfA, pch=3)

points(time, FeONPcLysConfB, pch=3)

points(time,FeONPcLysMean-FeONPcLysSD, pch=3)

points(time,FeONPcLysMean+FeONPcLysSD, pch=3)
```

References

1. Brust M, Walker M, Bethell D, Schiffrin DJ, Whyman R: **Synthesis of thiol-derivatised gold nanoparticles in a two-phase liquid–liquid system.** *J Chem Soc, Chem Commun* 1994, **801**.
2. Kaiser U, Jimenez de Aberasturi D, Malinowski R, Amin F, Parak WJ, Heimbrodt W: **Multiplexed measurements by time resolved spectroscopy using colloidal CdSe/ZnS quantum dots.** *Appl Phys Lett* 2014, **104:** 041901.
3. Kreyling WG, Abdelmonem AM, Ali Z, Alves F, Geiser M, Haberl N *et al.*: **In vivo integrity of polymer-coated gold nanoparticles.** *Nat Nanotechnol* 2015, **10:** 619-623.
4. Liu X, Atwater M, Wang J, Huo Q: **Extinction coefficient of gold nanoparticles with different sizes and different capping ligands.** *Colloids Surf B Biointerfaces* 2007, **58:** 3-7.
5. Hyeon T, Lee SS, Park J, Chung Y, Na HB: **Synthesis of highly crystalline and monodisperse maghemite nanocrystallites without a size-selection process.** *J Am Chem Soc* 2001, **123:** 12798-12801.
6. Lin CA, Sperling RA, Li JK, Yang TY, Li PY, Zanella M *et al.*: **Design of an amphiphilic polymer for nanoparticle coating and functionalization.** *Small* 2008, **4:** 334-341.
7. Pellegrino T, Manna L, Kudera S, Koktysh D, Rogach AL, Natile G *et al.*: **Hydrophobic nanocrystals coated with an amphiphilic polymer shell: a general route to water soluble nanocrystals.** *Nano Letters* 2004, **4:** 703-707.
8. Zhang F, Lees E, Amin F, Rivera GP, Yang F, Mulvaney P *et al.*: **Polymer-coated nanoparticles: a universal tool for biolabelling experiments.** *Small* 2011, **7:** 3113-3127.
9. Rothen-Rutishauser B, Kuhn DA, Ali Z, Gasser M, Amin F, Parak WJ *et al.*: **Quantification of gold nanoparticle cell uptake under controlled biological conditions and adequate resolution.** *Nanomedicine (London, UK)* 2013.
10. Yakovlev AV, Zhang F, Zulqurnain A, Azhar-Zahoor A, Luccardini C, Gaillard S *et al.*: **Wrapping nanocrystals with an amphiphilic polymer preloaded with fixed amounts of fluorophore generates FRET-based nanoprobes with a controlled donor/acceptor ratio.** *Langmuir* 2009, **25:** 3232-3239.
11. Sperling RA, Rivera GP, Zhang F, Zanella M, Parak WJ: **Biological applications of gold nanoparticles.** *Chem Soc Rev* 2008, **37:** 1896-1908.
